# Large-scale combinatorial optical barcoding of cells with laser particles

**DOI:** 10.1038/s41377-025-01809-x

**Published:** 2025-04-01

**Authors:** Nicola Martino, Hao Yan, Geoffrey Abbott, Marissa Fahlberg, Sarah Forward, Kwon-Hyeon Kim, Yue Wu, Han Zhu, Sheldon J. J. Kwok, Seok-Hyun Yun

**Affiliations:** 1 Harvard Medical School and Wellman Center for Photomedicine, Massachusetts General Hospital, Cambridge, MA 02139 USA; 2 Broad Institute of MIT and Harvard, Cambridge, MA 02142 USA; 3 LASE Innovation Inc., Waltham, MA 02451 USA; 4 Harvard-MIT Health Sciences and Technology, Massachusetts Institute of Technology, Cambridge, MA 02139 USA; 5 Present Address: Tsinghua Shenzhen International Graduate School, Tsinghua University, Shenzhen, 518055 China

**Keywords:** Biophotonics, Microresonators, Semiconductor lasers, Imaging and sensing

## Abstract

The identification of individual cells is crucial for advancements in single-cell analysis. Optically readable barcodes provide a means to distinguish and track cells through repeated, non-destructive measurements. Traditional fluorophore-based methods are limited by the finite number of unique barcodes they can produce. Laser particles (LPs), which emit narrowband peaks over a wide spectral range, have emerged as a promising technology for single-cell barcoding. Here, we demonstrate the use of multiple LPs to generate combinatorial barcodes, enabling the identification of a vast number of live cells. We introduce a theoretical framework for estimating the number of LPs required for unique barcodes and the expected identification error rate. Additionally, we present an improved LP-tagging method that is highly effective across a variety of cell types and evaluate its biocompatibility. Our experimental results show successful barcoding of several million cells, closely matching our theoretical predictions. This research marks a significant step forward in the scalability of LP technology for single-cell tracking and analysis.

## Abstract (web summary)

A combinatorial approach using laser particles for optical barcoding enables distinguishing thousands to millions of cells. Theoretical framework and enhanced tagging pave the way for scalable single-cell tracking and analysis.

## Graphical abstract

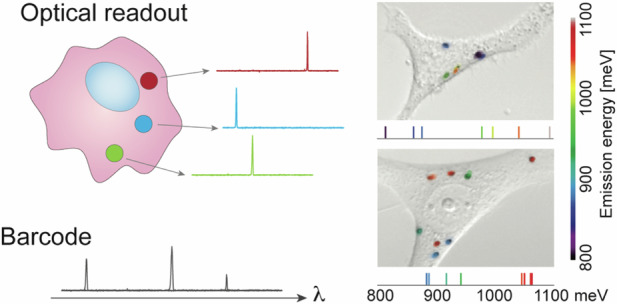

## Introduction

Single-cell analysis has established itself as a vital technique in life sciences and clinical medicine^1,2^. It goes beyond the limitations of conventional ensemble analysis of cell populations, allowing for the detailed investigation of individual cells^3^. Fluorescence microscopy permits the imaging of individual cells for complex functional^4–6^ and molecular^7–9^ assays. Flow cytometry and microfluidic analyses acquire cell samples with high throughput in a fluidic stream, sequentially identifying the phenotypes of individual cells^10,11^. Moreover, a variety of single-cell omics approaches, including droplet-based sequencing^12,13^ and spatial capture methods^14–17^, have been developed in recent years, allowing a more comprehensive understanding of cells’ molecular profiles.

In an ideal experimental workflow, one would simultaneously profile as many aspects of each individual cell as possible, including their lineage, dynamic molecular states, spatial positions, and interactions with the microenvironment^1^. Optical cell barcoding enhances this capability by marking each cell with unique, optically readable identifiers^18^, facilitating their easy recognition and examination via microscopes, flow cytometers, sequencers, and other optical instruments. This technique employs distinct, optically detectable labels to tag cells, akin to the use of Universal Product Codes for merchandise or name tags for individuals. The optical readout offers significant benefits, including real-time, non-contact, non-destructive, and repeatable cell identification. Importantly, optical barcoding enables the remote tracking of live cells in vivo and supports comprehensive cross-platform analyses. For instance, cells tagged and observed through time-lapse microscopy can subsequently be isolated and analyzed using flow cytometry or single cell sequencing^11,18^. The data acquired from these platforms can then be linked back to individual cells based on their unique barcodes, enabling holistic, multi-dimensional single-cell analysis.

While fluorescence barcoding has found application in labeling capture beads for multiplexed bead assays^19,20^, its extension to single-cell identification has been limited. The wide emission spectra of fluorophores and quantum dots constrain the number of unique tags to a few hundred at most^21,22^. Other approaches such as Raman multiplexed probes^23^ face the same limitations. Given that typical single-cell analyses necessitate between 10^4^ and 10^5^ barcodes per sample and even upwards of 10^6^ for rare cell types, the limitation in number of unique tags poses a bottleneck. Additionally, fluorophores play a crucial role in a broad spectrum of biological analyses, encompassing imaging, flow cytometry, and sequencing^24^. Therefore, there is an unmet need for a barcoding technology that can accommodate a larger number of cells and is compatible with existing fluorescence-based methods.

In the past decade, an innovative optical barcoding strategy utilizing narrowband-emission Laser Particles (LPs) has been developed^25–28^. Single-mode LPs exploit cavity-enhanced stimulated emission to produce sub-nanometer linewidths, substantially narrower than the emission bandwidths of traditional fluorophores. We have shown that semiconductor-based microdisk LPs can emit in a narrow band (< 0.4 nm) across the 1150–1650 nm spectrum^25,29^. With a binning of 1 nm, they provide 500 distinct colors. This technology enabled us to track thousands of cancer cells within a tumor spheroid model^25^. Despite this breakthrough, the available palette size is still not adequate for the unique barcoding of cells in many single-cell biology applications.

Using a combinatorial barcoding strategy can significantly extend the number of available barcodes beyond the limitations of this finite palette size. This method involves labeling each cell with several LPs, each characterized by distinct laser-emission peaks. The total number of unique barcodes can be estimated by the combination formula, $C(l,m)$, where*l*is the number of distinguishable laser colors, and *m* is the number of LPs per cell. With*l*typically on the order of a few hundreds, modest values of *m* = 3, 4 are enough to satisfy the uniqueness requirements in most relevant applications. This approach has been successfully applied to tag and analyze 10^5^ to 10^6^ human peripheral blood mononuclear cells using multi-pass flow cytometry^11^. Unlike combinatorial methods that rely on different fluorophore intensity levels^21,30^, LP barcoding benefits from its purely spectral encoding with enhanced robustness against variability in tagging concentrations and signal attenuation.

In this study, we undertake both theoretical and experimental analyses of this combinatorial LP-barcoding strategy. Our work encompasses the development of a theoretical framework to estimate the error rate associated with duplicate barcodes and noise in spectral reading, which is contingent upon specific experimental conditions, such as the number of LPs per cell and total number of analyzed cells. We further analyze the efficiency of advanced tagging protocols designed to enhance the efficiency of labeling cells with multiple LPs, validating their efficacy across both adherent and suspension cell types. Our methodology is rigorously tested by measuring the optical barcodes of extensive cohort of cells, exceeding 1 million, under realistic experimental conditions. Moreover, we assess the biological effects of LP tagging on cell division and gene expression for cancer cells, demonstrating the potential of this technology for broad applications in cellular biology and medical diagnostics. This work lays the foundation for the widespread use of LP barcoding in multidimensional live-cell analysis of massive numbers of cells.

## Results

### Combinatorial barcoding with discrete spectral elements

Combinatorial barcoding involves physically associating a set of LPs with a cell. LPs can either be internalized into the cytoplasm or attached to the cell membrane’s exterior, depending on the LP’s size, surface properties, cell types and their states. We assume that all LPs operate on a single laser mode, meaning the barcode’s multiplicity corresponds to the number of LPs associated with a cell, denoted by *m*. The implications of dual-mode lasing will be addressed subsequently. The multiplicity, *m*, may differ among cells. Our analysis focuses on barcoding with specific values of *m* from 1 to 5. These findings will later be extended to scenarios involving groups of cells tagged with varying *m* values, such as through stochastic tagging.

**Fig. 1:**
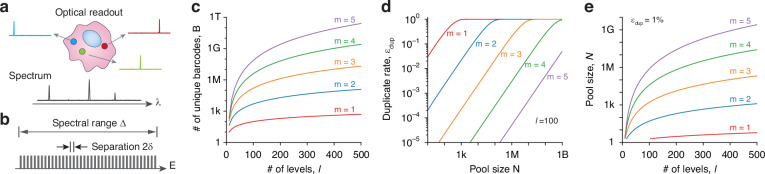
Combinatorial barcodes based on discrete energy levels. **a** Illustration of a cell tagged with three spectrally distinct LPs (circles). **b** Schematic of the barcoding strategy utilizing discrete energy levels. **c** Total number of unique barcodes, given by $\sim {l}^{m}/m!$. **d** Barcode duplicate rates across varying pool sizes (number of cells) for *l* = 100. **e** Maximum possible pool size achievable with a duplicate rate of 1%

Figure 1a illustrates a cell tagged with three LPs. With optical pumping above the lasing threshold, each LP emits a characteristic narrowband emission spectrum. Together, these peak wavelengths form the cell’s optical barcode. Considering that spectral linewidths and measurement noise are relatively constant in frequency rather than wavelength, we convert wavelength to photon energy (*E*) for convenience. Since the order of the emission lines cannot be differentiated, each barcode is represented as a sorted list of energies, ${\boldsymbol{E}}=({E}_{1},\,\ldots \,{E}_{m})$, where ${E}_{j}\, >\, {E}_{i}$ for all $j\, >\, i$. Consider the scenario that the possible laser spectral lines are discrete, and there are *l* possible levels (colors or spectral bins). This setup is shown in Fig. 1b, where the total spectral range is Δ, emission energies are separated by a gap of 2${\rm{\delta }}$, and $l=\Delta /2{\rm{\delta }}$. The total count of unique barcodes, $B$, is determined by the binomial coefficient $C(l,m)$. This calculation is depicted in Fig. 1c across a variety of $l$ and *m* values. Since $l\gg m$,

$$B\approx \frac{{l}^{m}}{m!}=\frac{1}{m!}{\left(\frac{\Delta }{2\delta }\right)}^{m}$$

When LPs are randomly allocated to cells, the total count of unique barcodes, *B*, doesn’t equate to the total number of cells that can be uniquely identified because a specific barcode might be assigned more than once. With the assumption that all energy levels have an equal probability of occurrence, for a given pool size *N* (representing the number of tagged cells), the probability of barcode duplication, ${\varepsilon }_{{\rm{dup}}}$, can be calculated as follows:

1 $${\varepsilon }_{{\rm{dup}}}=1-{\left(1-\frac{1}{B}\right)}^{N-1}\approx \frac{m!}{{l}^{m}}N$$

where $B\gg N$ has been used for the approximation. Figure 1d shows the duplicate rate as a function of the pool size, with *l* = 100. This result is valuable for experiment planning, particularly when aiming to keep the duplicate rates below a certain threshold. According to Eq. (1), to tag *N* = 10^4^ cells while maintaining a duplicate rate below 1%, a minimum of *B* = 10^6^ unique barcodes is necessary. Figure 1e outlines this requirement as a function of *l*, providing a practical guideline to achieve desired levels of barcode uniqueness.

### Combinatorial barcoding with continuous spectral elements

**Fig. 2:**
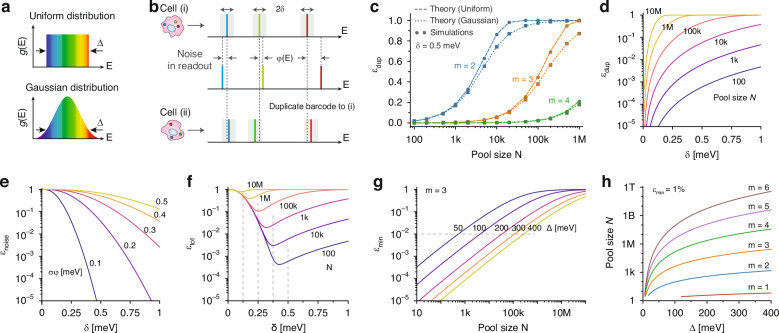
Combinatorial barcoding using continuous energy levels. **a** Uniform and Gaussian distributions for LP emission. **b** Measurement noise and duplication events. **c** Theoretical duplicate rates (curves) and computer-simulation results (markers) for both uniform and Gaussian distributions ($\Delta =$100 meV and $\delta =$0.5 meV). **d** Duplicate rates as a function of gap size $\delta$ across pool sizes. **e** Noise-induced error rates versus $\delta$ for various ${\sigma }_{\varphi }$. **f** Total error rates for diverse sample sizes, indicating ${\delta }_{{opt}}$ and ${\varepsilon }_{\min }$ at each curve’s minimum. **g** Minimum total error rate relative to sample size for varying Δ, with a dashed line marking ${\varepsilon }_{\min }=$1%. **h** Maximum sample size for achieving ${\varepsilon }_{\min }=$1%. All analyses assume a uniform distribution, $\Delta =$100 meV, $m=$3, and ${\sigma }_{\varphi }=$0.1 meV, unless noted otherwise

In this section, we explore the scenario where the photon energies of LPs span a continuous spectrum rather than discrete levels. Building on the discrete model, we employ a probability density function, $g\left({\rm{E}}\right)$, to describe the distribution of LP emission peaks, where $\int g\left(E\right){dE}=1$. We examine two distinct probability functions: the *uniform* distribution, which models LPs created from the various semiconductor blends, and the *Gaussian* distribution, representing the typical distribution from a single semiconductor type^25,29,31^. To compare the spectral ranges, we introduce a width parameter Δ (as depicted in Fig. 2a). In the uniform case, $\Delta$ spans the entire spectral range (matching $\Delta$ from Fig. 1b). For the Gaussian distribution, we define $\Delta =\sqrt{12}{\rm{\sigma }}$, with σ the standard deviation. This ensures that both distributions have the same standard deviation, facilitating a comparison. When selecting $m$ LPs from a common pool, their combined probability distribution, ${G}_{m}\left({\boldsymbol{E}}\right)$, is defined as

$${G}_{m}\left({E}_{1},\ldots ,{E}_{m}\right)=m!{\prod }_{{\rm{i}}=1}^{m}g({E}_{i})$$

For every barcode measurement there may be slight variations in the energy lines due to experimental conditions or noises such as optical and electrical fluctuations, temperature differences, or spectrometer miscalibrations. These variations, collectively referred to as ‘noise’, are depicted in Fig. 2b. Therefore, it is necessary to employ a metric to evaluate the similarity between pairs of measured barcodes. Although this issue exists in discrete-level barcoding, choosing a sufficiently large gap $2{\rm{\delta }}$ can mitigate the effect of noise. To address noise in the continuous energy spectrum, we conceptualize an *m*-dimensional energy space, with each measurement represented as a point within this space. We set a threshold $\delta$: two measurements ${{\boldsymbol{E}}}_{A}$ and ${{\boldsymbol{E}}}_{B}$ are considered identical if their distance in this space is less than $\delta$, assuming both have the same multiplicity $m$. Measurements of different *m* values are presumed to originate from distinct barcodes, although real measurements might not always reflect this due to non-idealities in the barcode reading process. We adopt the Chebyshev metric to define the distance between ${{\boldsymbol{E}}}_{A}$ and ${{\boldsymbol{E}}}_{B}$ as $\mathop{\max }\limits_{i=1,\ldots ,m}\left|{E}_{A,i}-{E}_{B,i}\right|$. This metric designates a (hyper)cube of side length $2\delta$ around each barcode in the $m$-dimensional space. The formula for the duplicate rate is as follows (see Supplementary Note A):

2 $${\varepsilon }_{{\rm{dup}}}=1-\int {G}_{m}\left({\boldsymbol{E}}\right){\left(1-{\left(2{\rm{\delta }}\right)}^{m}{G}_{m}\left({\boldsymbol{E}}\right)\right)}^{N-1}{\rm{d}}{\boldsymbol{E}}$$

For the uniform distribution, the duplicate rate simplifies to ${\varepsilon }_{{\rm{dup}}}\mathop{\to }\limits_{{\rm{uniform}}}1-{\left(1-\frac{1}{{B}_{{\rm{eff}}}}\right)}^{N-1}$, where ${B}_{{\rm{eff}}}=\frac{1}{m!}{\left(\frac{\Delta }{2\delta }\right)}^{m}$. This equation mirrors Eq. 1, except here $B$ is substituted with ${B}_{{\rm{eff}}}$, representing the effective number of unique barcodes in the continuous case. This effective count is given by the ratio of the total barcode space to the volume occupied by a single barcode. For the Gaussian distribution, ${\varepsilon }_{{\rm{dup}}}$ can be calculated by numerical integration. The estimated duplicate rates for a spectral width $\Delta =$ 100 meV and a threshold $\delta =$ 0.5 meV are depicted in Fig. 2c for both uniform and Gaussian distributions, respectively, and in Fig. 2d for different values of δ. To validate Eq. 2, computer simulations were performed, randomly selecting *N* samples from the defined distribution ${G}_{m}\left({\boldsymbol{E}}\right)$ and tallying duplicates using the Chebyshev metric. The simulation outcomes align closely with the theoretical prediction (Fig. 2c). Notably at low duplicate rates (less than 10%), the discrepancies between the uniform and Gaussian cases are minimal.

While aiming for a smaller $\delta$ is advantageous to reduce duplicate rates, it increases the likelihood of error in barcode identification due to measurement noise. The probability function $\varphi (E^{\prime} )$ denotes the likelihood of a measurement yielding a fluctuation of $E^{\prime}$ with respect to a mean value. For normal distribution noise with standard deviation ${\sigma }_{\varphi },\varphi \left(E^{\prime} \right)=\frac{1}{{\sigma }_{\varphi }\sqrt{2\pi }}\exp (-\frac{{E}^{{\prime} 2}}{2{\sigma }_{\varphi }^{2}})$. The probability that two measurements of the same barcode are separated by more than $2\delta$, leading to a mismatch, is expressed as (see Supplementary Note B):

3 $${\varepsilon }_{{\rm{noise}}}=1-{\left({\int }_{\!-{\rm{\delta }}}^{{\rm{\delta }}}\varphi \left({E}^{{\prime} }\right){\rm{d}}{E}^{{\prime} }\right)}^{\!\!m}$$

The noise-induced error ${\varepsilon }_{{\rm{noise}}}$ is illustrated in Fig. 2e for different ${\sigma }_{\varphi }$ values. An optimal $\delta$ can be determined, which balances the trade-off between maximizing the count of unique barcodes and minimizing duplication and noise-induced error. Figure 2f presents the compounded error rate, ${\varepsilon }_{{\rm{tot}}}$, calculated as

$${\varepsilon }_{{\rm{tot}}}={\varepsilon }_{{\rm{dup}}}+{\varepsilon }_{{\rm{noise}}}-{\varepsilon }_{{\rm{dup}}}{\varepsilon }_{{\rm{noise,}}}$$

for a scenario where ${\sigma }_{\varphi }=0.1{\rm{meV}}$. The optimal threshold, ${\delta }_{{opt}}$, corresponds to the point where the total error rate ${\varepsilon }_{{\rm{tot}}}$ is minimized. Below this optimum, noise-induced errors escalate sharply as $\delta$ diverges from ${\delta }_{{opt}}$. As anticipated, ${\delta }_{{opt}}$ diminishes as the sample size $N$ increases. For smaller pools of samples, a larger $\delta$ is preferable to ensure robust identification against noise. Conversely, a smaller $\delta$ becomes more desirable for larger sample sizes to reduce the likelihood of duplicate identifications. Figure 2g illustrates the minimum total error rate, ${\varepsilon }_{\min }$, achieved at $\delta ={\delta }_{{opt}}$, across varying pool sizes and as a function of the bandwidth $\Delta$.

Estimating the maximum permissible sample size *N* (number of cells) that maintains the total error rate, ${\varepsilon }_{{\rm{tot}}}$, below a specific threshold suitable for a particular application, ${\varepsilon }_{0}$, can be highly beneficial. As an example, Fig. 2h explores this for ${\varepsilon }_{0}=$1%, demonstrating how the maximum $N$ varies with $\Delta$ and $m$, or the necessary $m$ value for accommodating a given sample size. In this case, with a $\Delta =$300 meV covering a spectral range of 1150–1600 nm, utilizing 3 or 4 LPs per cell could effectively barcode between 10^4^ and 10^6^ cells, providing a scalable solution for reliable cell tagging across a broad spectrum of applications.

### Combinatorial barcoding with Poisson distribution multiplicity

**Fig. 3:**
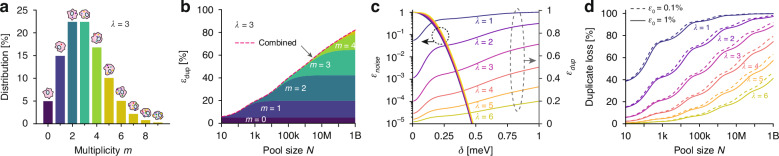
Samples containing different multiplicities. **a** The Poisson distribution for the number of LPs per cell (multiplicity *m*) with an average tagging ratio of $\lambda =3$. **b** Relative contributions to the duplicate rate from barcodes of varying multiplicities for $\lambda =3$. **c** ${\varepsilon }_{{\rm{noise}}}$ and ${\varepsilon }_{{\rm{dup}}}$ across various $\lambda$ values for a sample size of $N=100{\rm{k}}$. **d** The duplication-induced barcode loss as a function of sample size under different Poisson tagging ratios, based on $\Delta =100{\rm{meV}}$ and ${\sigma }_{\varphi }=0.1{\rm{meV}}$. The solid and dashed lines represent maximum acceptable error rates of 1% and 0.1%, respectively

In practice, the number of LPs acquired by cells can vary, leading to different multiplicities within a sample pool^11,25^. For a purely stochastic tagging process, the number of LPs per cell is expected to follow a Poisson distribution. Figure 3a illustrates an example of this distribution with an average tagging ratio ($\lambda$) of 3 LPs per cell. Here, the duplicate rate for each multiplicity level is calculated as previously described, and the total duplicate rate is obtained by summing these rates, weighted by their respective proportions. Figure 3b presents results for a uniform spectral distribution with $\Delta =$100 meV, highlighting contributions from subgroups with varying multiplicities. Upon measuring the entire sample pool, duplicates can be detected and excluded from analysis, thereby treating duplication events as sample loss rather than a barcoding error. However, mismatches due to noise are generally indistinguishable and must be acknowledged as inherent errors. Figure 3c shows ${\varepsilon }_{{\rm{dup}}}$ and ${\varepsilon }_{{\rm{noise}}}$ across different tagging ratios $\lambda$, for $N=$100k. By identifying duplicates, it is possible to select a $\delta$ value that aligns ${\varepsilon }_{{\rm{noise}}}$ with the maximum acceptable error rate ${\varepsilon }_{0}$. At this optimal $\delta$, the proportion of duplicate barcodes (${\varepsilon }_{{\rm{dup}}}$) can be considered as sample loss. Figure 3d depicts the impact of different tagging ratios on duplicate loss, indicating that a higher tagging ratio reduces duplication loss for a specified sample size.

### Efficient protocols for stochastic tagging

**Fig. 4:**
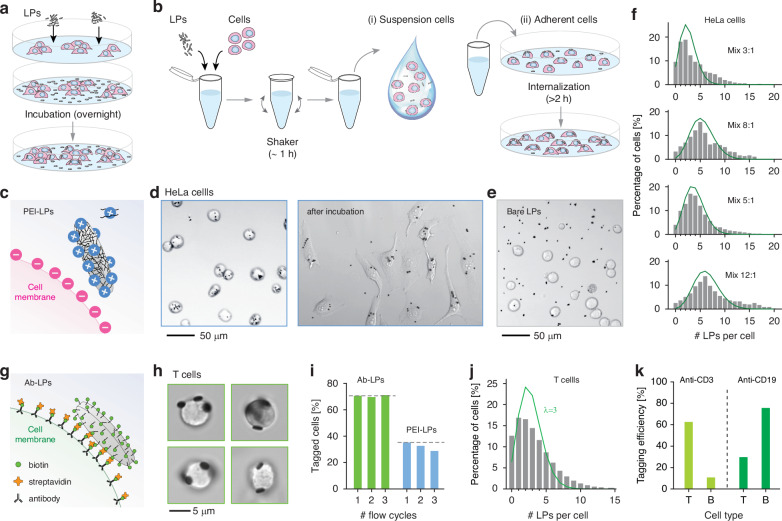
Protocols for efficient combinatorial tagging. **a** Conventional method schematic: LPs are added to a cell culture on a plate. **b** Revised method schematic: Cells mixed with LPs in solution, then transferred to (i) a tube for suspension cells or (ii) a culture dish for adherent cells. **c** PEI-LP tagging schematic. **d** Brightfield images of HeLa cells after mixing with PEI-LPs immediately after plating (left) and after 24-h incubation (right). **e** Brightfield image of HeLa cells after incubation with bare silica-coated LPs without PEI coating. **f** Histogram of PEI-LPs per HeLa cell for various initial mixing ratios (LP-to-cell), measured 24 h post-plating (*N* = 366, 375, 370, and 471 cells, respectively, from top to bottom). **g** Antibody-mediated tagging schematic. **h** Images of human T cells tagged with anti-CD3 LPs. **i** A comparison of tagging efficiency and stability between anti-CD3 and PEI functionalization for T cells, defined as the presence of at least one LP per cell, measured across three consecutive runs through the flow cell of a commercial flow cytometer. **j** Histogram of anti-CD3-LPs on T cells for a 5:1 mixing ratio (330,000 cells). **k** Tagging percentage for T and B cells with anti-CD3-biotin (left) or anti-CD19-biotin (right), demonstrating selective tagging of CD3^+^ T cells and CD19^+^ B cells

Our initial approach for tagging cancer cells with LPs involved co-culturing cells alongside LPs in a dish^25^ (Fig. 4a). However, this strategy often led to uneven tagging, leaving many untagged cells even when using high LP-to-cell ratios (Supplementary Fig. S1). The tendency of LPs to quickly settle at the dish bottom reduced the opportunities for cells to interact with LPs, resulting in variable tagging efficiency depending on cell concentration, motility, and incubation duration. Additionally, for cells growing in clusters tagging is typically confined to peripheral cells, with central cells remaining largely untagged.

In our recent work with blood cells^11^, we have developed a tagging protocol that involves mixing cells with LPs in a sample tube (Fig. 4b). This approach ensures a uniformly high probability for each cell to encounter LPs, achieving stochastically uniform and improved tagging efficiency. This technique is particularly effective for tagging immune cells in blood samples that are maintained in suspension. In this work, we extended this approach to adherent cells (Fig. 4b, ii). To enhance the binding of LPs to cell membranes during mixing, we developed two surface-functionalization strategies for LPs^32^. The first uses cationic polymers, such as polyethylenimine (PEI) and polylysine^33,34^, facilitating attachment to negatively charged cell membranes through non-specific electrostatic interactions. The second strategy relies on antibodies to achieve either cell-type-specific attachment or broad tagging by targeting ubiquitous surface receptors. This is achieved by tagging cells with biotinylated antibodies and using biotinylated LPs, linking the two via streptavidin in a similar configuration to standard “sandwich” assays^35^. Antibody-functionalized microlasers have been recently demonstrated for local antigen detection via refractive-index sensing^36^.

To test both methods for various cell types, we prepared LPs from six different InGaAsP compositions^25^, spanning a wide spectral range of 800–1100 meV (∆ = 300 meV). The particles’ diameters ranged from 1.6 to 1.8 µm, with thicknesses varying between 220 and 290 nm. The semiconductor particles were encapsulated in a protective silica layer either 30–50 nm or 100 nm thick before being functionalized with either PEI or biotin (see Methods).

PEI-coated LPs are compatible with a broad spectrum of cell types, as their attachment relies on electrostatic attraction between the negatively charged plasma membrane of cells and the positively charged polymer branches^37^ (Fig. 4c). All adherent cell types investigated tended to internalize LPs following their attachment to the cell membrane, typically within a few hours after plating. Tagging with PEI-LPs was efficient for a variety of adherent cells, including HeLa, MCF7, and 4T1 cancer cell lines, L929 fibroblasts, Raw264.7 macrophages, and mouse cortical neural stem cells (Supplementary Fig. S2). Confocal 3D microscopy confirmed the internalization (Supplementary Fig. S3). Figure 4d shows HeLa cells tagged with a 5:1 LP-to-cell mixing ratio, immediately after plating on a culture dish (left) and following incubation (right). The majority of cells exhibited multiple LPs localized within their cytoplasm. Only a few unbound LPs were visible in the extracellular space. In contrast, bare silica-coated LPs without PEI failed to attach to the cell membrane and remained in the culture medium, as shown in Fig. 4e. Different LP-to-cell mixing ratios resulted in various average tagging ratios (Fig. 4f and Supplementary Fig. S4). The distributions of tagged LP numbers approximately followed Poisson statistics, although some deviations are observed especially at higher multiplicities. Similar tagging efficiencies were obtained for MCF7 cells (Supplementary Fig. S5).

For antibody-mediated tagging, we used LPs functionalized with biotin (see Methods). These LPs were added to cells pre-coupled to biotinylated antibodies and mixed with streptavidin (Fig. 4g). Anti-CD3 antibodies were chosen for T-cell tagging. Figure 4h displays images of human T-cells tagged with anti-CD3 LPs, revealing LPs attached to the cell membrane after 24 h of incubation. Generally, blood cells resist LP uptake, except for activated monocytes and macrophages. Despite the LPs remaining external, the biotin-streptavidin bond ensured durable tagging, resistant to pipetting and multiple flow cytometry runs. Figure 4i shows LP retention for T cells tagged with anti-CD3-LPs and PEI-LPs at an identical LP-to-cell mixing ratio of 5:1. PEI-LPs exhibited a lower initial tagging efficiency and a modest yet noticeable decline in retention across multiple runs through the flow cell of a commercial hydrodynamic-focusing flow cytometer (Cytoflex S, Beckman Coulter). In contrast, anti-CD3-LPs showed higher initial tagging efficiency and sustained consistent attachment, attributed to the robust biotin-streptavidin interaction. Figure 4j presents a typical LP number histogram for a 5:1 LP-to-cell mixing ratio, indicating a Poisson-like distribution. Their application to human peripheral blood mononuclear cells (PBMCs) with anti-CD3 biotin showed that T lymphocytes (CD3^+^) had substantially higher tagging efficiency compared to B lymphocytes (CD19^+^). Conversely, employing anti-CD19-biotin preferentially tagged CD19^+^ B cells over T cells (Fig. 4k and Supplementary Fig. S6). Mixing these two antibodies in suitable ratios could achieve uniform tagging efficiency across both T and B cells. Additionally, the antibody approach proved effective for other cell types, such as mouse splenocytes with anti-H-2Kd-biotin with anti-CD45-biotin, and human leukemia cells (KOPN-8) with anti-β_2_M-biotin (Supplementary Fig. S7).

### Experimental validation of large-scale combinatorial barcoding

Optical barcodes can be measured via both LP-reading (LASE) laser-scanning confocal microscopy^25,38^ and LASE flow cytometry^11^. While supporting conventional fluorescence-based measurements, each instrument incorporates a 1064 nm nanosecond pump laser (2-MHz pulse repetition rate) and a high-resolution spectrometer using a diffraction grating and a linear InGaAs array camera (76 or 147 kHz readout rate).

**Fig. 5:**
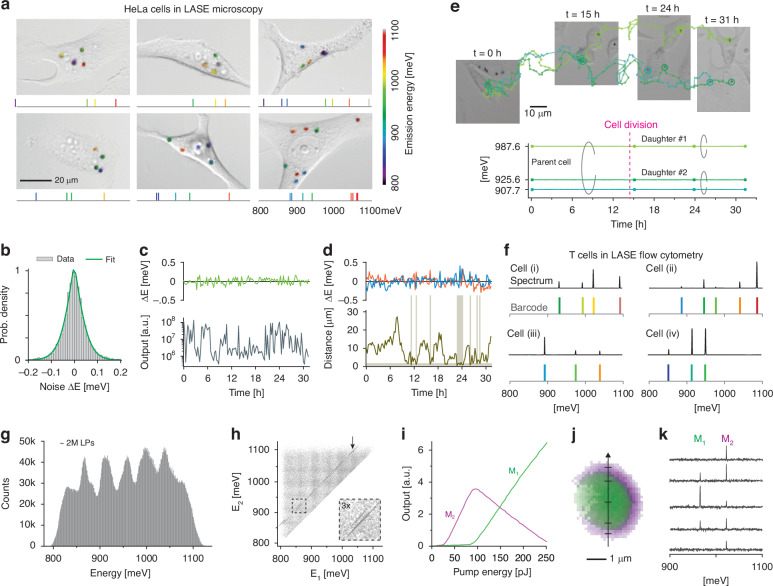
Experimental validation of combinatorial barcoding. **a** Brightfield images showcase six HeLa cells with overlaid laser emissions from LPs, color-coded by photon energy, where transparency indicates signal intensity per pixel. Below each image, the corresponding optical barcodes are presented. **b** The noise probability density function measured from 156 LPs. The green curve represents the best fit to a generalized normal function (*α* = 0.047 meV, *β* = 1.28). **c** LP emission energy (top) and intensity (bottom) of an LP in a HeLa cell. **d** The emission energies of two LPs within a HeLa cell (top) as their distance varies (bottom). Grey shaded areas indicate time intervals when the two LPs are in physical contact. **e** Tracking a single HeLa cell from time 0 and its daughter cells after division at 14 h. (Top) Representative brightfield images of the cells and their trajectories (lines). (Bottom) Spectral peaks measured over 31 h. **f** Spectra and barcodes of four T cells as recorded by LASE flow cytometry. **g** Distribution of emission line energies from LP-tagged T cells. **h** Correlation plot between pairs of barcode energies, ${E}_{1}$ and ${E}_{2}$, in the dataset for *m* = 2. Most barcodes display random ${E}_{1}$ and ${E}_{2}$ values, with the exception of a subset represented as diagonal streaks (arrow), indicating strong correlation characteristic of dual-mode lasing. Inset, zoom-in view of the dashed box region. **i** Output power of two modes, M_1_ and M_2_, from a single LP as a function of pump energy. **j** Spatial mapping of a dual-mode LP, achieved by scanning the confocal pump beam focus over the LP. **k** Individual spectra from the LP in (**j**) at various marked locations across its diameter

Using the microscope, we captured bright-field images of LP-tagged HeLa cells, with laser emission profiles from individual LPs superimposed, and photon energy color-coded. Figure 5a features six representative cells showcasing this photon energy barcoding. To characterize the system’s noise function $\varphi \left({E}^{{\prime} }\right)$, we recorded the emission spectra of LPs over 50 min at 45 s intervals, yielding 71 data points per LP. Figure 5b presents a histogram of these energy fluctuations, averaged across 156 LPs (Supplementary Fig. S8). This histogram was modeled using a generalized Gaussian distribution function, $\varphi \left({E}^{{\prime} }\right)=\frac{\beta }{2\alpha \Gamma (1/\beta )}{e}^{-{\left(\left|{E}^{{\prime} }\right|/\alpha \right)}^{{\rm{\beta }}}}$, with an effective standard deviation *α* = 0.047 meV and an exponent *β* = 1.28. This model, capturing the Lorentzian-like tails of the distribution more accurately than a standard Gaussian function, allowed us to estimate the noise-induced error rates, ${\varepsilon }_{{\rm{noise}}}$, for various $\delta$ values (Supplementary Fig. S9).

To evaluate the stability of LP barcodes, we recorded confocal images of HeLa cells every 15 min over a 31 h period. Figure 5c shows the output intensity of a representative LP over time (bottom) and the variations in its spectral peak energy (top). The observed intensity fluctuations arise from changes in the LP’s orientation, where its whispering gallery (WG) mode emission predominantly lies in the in-plane direction^39^. Despite intensity fluctuations spanning over two orders of magnitude, the spectral energy remained stable within ±0.05 meV. We also analyzed cells containing multiple LPs to assess the effects of physical interactions between LPs within the cytoplasm. Figure 5d shows the time-lapse spectral peak profiles of two LPs within a single cell (top), alongside the distance between the two LPs measured from confocal images. Time intervals during which the two LPs were in contact (distance < 2 µm) are highlighted as gray bands. Compared to single LPs, the spectral fluctuations of individual LPs in pairs showed a modest increase but remained within ±0.07 meV. The 100-nm-thick silica coating was crucial in maintaining this stability (Supplementary Fig. S10).

Figure 5e illustrates time-lapse images of a HeLa cell undergoing division at approximately 14 h. The parent cell was initially tagged with three LPs with distinct lasing wavelengths. Following cell division, one LP was inherited by one daughter cell, while two were distributed to the other, as indicated in the spectral traces (Fig. 5e, bottom). Although this data demonstrates the potential for tracking cell lineage using multiple LPs, it also highlights an inherent limitation of LP barcodes: dilution upon mitosis. This process reduces LP multiplicity per cell (Supplementary Fig. 11) and decreases the number of unique signatures, making long-term tracking of proliferating cells challenging. Possible solutions to address this limitation will be discussed in Section *Combinatorial multiplet LPs*.

Next, we analyzed the barcodes of two million LP-tagged T cells using flow cytometry (Fig. 5f). Figure 5g shows the histogram of LP emission energies, reflecting the probability density $g\left(E\right)$ for our experimental LPs across over 300 meV. This profile, characterized by six broad envelopes, stems from the six semiconductor compositions in the LP pool. The WG modes in some LPs may lead to dual-mode lasing with close thresholds, producing two distinct energy peaks. To isolate them, we looked at barcodes with two lines from the dataset. Figure 5h reveals the energy correlation between these two peaks, identifying specific highly-correlated subsets (arrow) with spacing ranging from 50 to 70 meV, while the rest of barcodes have random correlation between ${E}_{1}$ and ${E}_{2}$. The interval aligns with the expected free spectral range for diameters of 1.6–1.8 µm. We thus infer that this subset represents cells tagged with one dual-mode LPs. We found that 7.1% of cells labeled as *m* = 2 were tagged with a single dual-mode LP. This population fraction is denoted ${f}_{{dm}}$ for subsequent reference.

Since dual-mode peaks are correlated, they do not contribute to barcoding in the same manner as two independent single-mode LPs. However, dual-mode lines are not equivalent to a single line, because the correlation or free-spectral range between the two modes depends on the resonator size and shape. This property could make a dual-mode LP slightly more effective than a single-mode LP, provided the dual modes can be consistently generated and measured. In practice, however, achieving stable dual-mode lasing is challenging. This is illustrated in Fig. 5i, which depicts the intensities of dual modes as a function of pump energy. One mode (M_2_) is detectable at lower pump powers, while the other mode (M_1_) becomes dominant at higher pump powers. This mode competition introduces significant variations in the output spectrum, increasing the risk of barcode misidentification, especially in single-shot measurements. This issue could be mitigated by continuously acquiring output spectra while scanning a pump beam across LPs, as in confocal microscopy, or by allowing LPs to flow through a stationary pump beam, as in flow cytometry. The typical Gaussian intensity profile of the pump beam enhances the likelihood of detecting both modes. This is demonstrated in Fig. 5j, k, where raster-scanning the confocal pump beam across the LP enables detection of M_1_, M_2_ or both modes, identifying the LP as a dual-mode resonator.

**Fig. 6:**
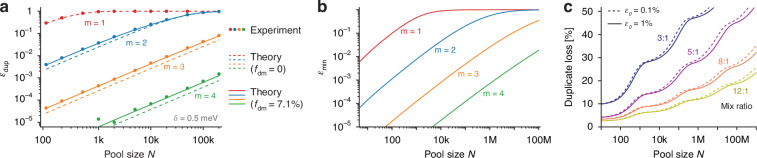
Comparison of experimental and simulation barcoding performance. **a** Duplicate rates for various multiplicities within the experimental pool. Circles represent experimental data; dashed lines denote the theoretical model based solely on single-mode LPs, while solid lines adjust for dual-mode LPs. **b** Theoretically calculated minimum total error rates for different multiplicities. **c** Expected duplicate losses for HeLa cells with tagging profiles as seen in Fig. 4f, at total error tolerance levels of 0.1% (dashed lines) and 1% (solid lines)

From the T-cell dataset, we identified duplicate barcodes within subsets of identical multiplicity. To simulate varying sample sizes ($N$), we randomly selected subsets from the complete dataset for analysis, depicted as circles in Fig. 6a. For comparison, theoretical duplicate rates (${\varepsilon }_{{\rm{dup}}}$) were computed using the observed $g\left(E\right)$ and Eq. (2). Initially, we utilized ${G}_{m}\left({\boldsymbol{E}}\right)=m!{\prod }_{{\rm{i}}=1}^{m}g({E}_{i})\equiv {G}_{{sm}}\left({\boldsymbol{E}}\right)$, assuming all LPs emitted a single mode from the histogram of Fig. 5g. While these calculations, shown as dashed curves in Fig. 6a, reasonably follow the experimental outcomes, they consistently underestimated the duplicate rate by about 60% for ${\varepsilon }_{{\rm{dup}}}$ less than a few percent. This discrepancy is linked to the inclusion of dual-mode LPs, whose correlated emission lines reduce the total number ($B$) of distinct barcodes. To account for the impact of dual-mode LPs, which represent 7.1% (${f}_{{dm}}$) of the pool, we adjusted the barcode distribution model:

$${G}_{m}\left({\boldsymbol{E}}\right)=\left(1-{f}_{{dm}}\right){G}_{{sm}}\left({\boldsymbol{E}}\right)+{f}_{{\!dm}}{G}_{{dm}}\left({\boldsymbol{E}}\right)$$

Here, ${G}_{{dm}}({\boldsymbol{E}}$) denotes the probability distribution for dual-mode LPs, derived from the identified correlation subset in Fig. 5h. We assumed that the number of barcodes containing multiple dual-mode LPs was negligible at low multiplicities given that ${f}_{{dm}}\ll 1$. Incorporating this adjusted model into Eq. 2 yielded a close match between theoretical predictions and experimental findings (Fig. 6a). Using the framework from Section *Combinatorial barcoding with continuous spectral elements* with experimentally derived duplicate rates and noise, we calculated the minimum total error rates (${\varepsilon }_{\min }$) achievable, as shown in Fig. 6b, and determined the optimal binning threshold (${\delta }_{{\rm{opt}}}$) (Supplementary Fig. S12). Lastly, applying the experimentally measured tagging distribution of HeLa cells (Fig. 4f), we evaluated duplicate-induced losses, with results for error tolerances (${\varepsilon }_{0}$) of 0.1% and 1% shown in Fig. 6c. For a 12:1 mix ratio ($\lambda \approx$ 6), we could limit duplicate-induced cell loss to less than 20% for pool sizes up to 100 M.

**Fig. 7:**
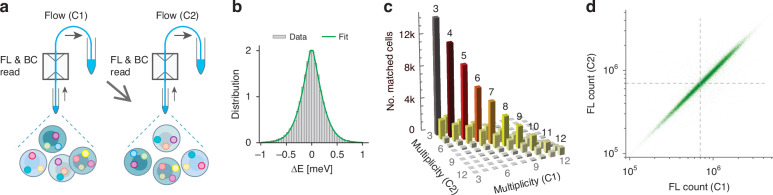
Matching HeLa cells across two flow cytometry measurements. **a** Schematic representation of the 2-run flow cytometry matching experiment. **b** Energy noise in the emission line measurements from the flow cytometry experiment. The green curve represents the best fit to a generalized Gaussian function with *α* = 0.25 meV and *β* = 1.37. **c** Comparison of barcode multiplicities for matched cells between the two flow cytometry runs, C1 and C2. **d** Scatter plot showing the correlation of fluorescence intensities for matched cells between the two runs

To assess the robustness of our combinatorial barcoding strategy in accurately identifying cells across different measurements, we performed a matching experiment using HeLa cells stained with a cytoplasmic fluorescent dye (CellTracker Green) and tagged with PEI-LPs at a 5:1 mixing ratio. Of 187,000 cells analyzed, approximately 118,000 cells (63%) were found to contain three or more LPs (3+ LPs). As illustrated in Fig. 7a, both the fluorescence intensity and laser spectra of the cells were measured in a first run (C1) using the LASE flow cytometer. The sample was then centrifuged, resuspended in fresh buffer and measured again in a second run (C2) using the same instrument. The C2 measurement identified 107,000 cells with 3+ LPs, reflecting a 10% cell loss during collection and centrifugation. Barcode matching between C1 and C2 datasets was performed based on their measured spectral peaks. While the theorical framework discussed in earlier sections considered matches only between identical multiplicities, our analysis allowed for matches between barcodes with different numbers of lines to account for practical deviations (see Methods). The measured energy noise (Fig. 7b) was characterized by *α* = 0.25 meV and *β* = 1.37, higher than previously reported values (Fig. 5b). The difference is attributed to different optical configurations of the instruments and the fact that the confocal microscope collects multiple spectra per LP, enabling more precise estimation of the central emission line, whereas high-speed flow cytometry records only a single spectrum per cell.

Despite this, the analysis successfully matched 99,900 cells (93.3%) between C1 and C2. Figure 7c presents a histogram of barcode multiplicities for the matched cells. Most matches occurred between barcodes of identical multiplicities, but some off-diagonal matches were observed, which are relatively symmetrically distributed. These discrepancies are attributed incomplete barcode detection during individual measurements. To further validate matching accuracy, the fluorescence intensities of the cytoplasmic dye were compared for each matched cell pair. As shown in the scatter plot in Fig. 7d, a strong correlation between the fluorescence intensities confirms the high fidelity of barcode detection and matching across repeated measurements.

### Combinatorial multiplet LPs

**Fig. 8:**
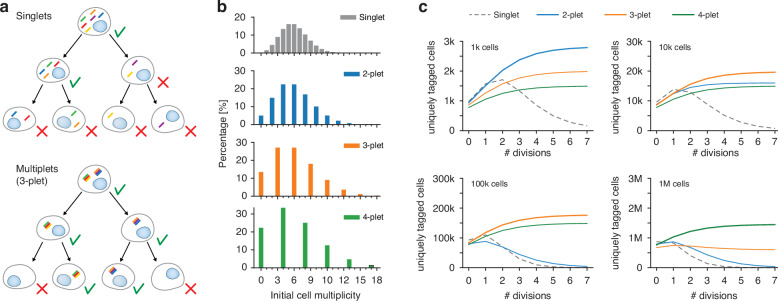
Simulation of multiplet tagging for reliable cell identification across proliferation. **a** Schematic comparison of singlet versus multiplet tagging and their inheritance patterns through successive cell divisions. **b** Initial distributions of barcode multiplicities for singlets, 2-plets, 3-plets, and 4-plets, all with an average LP number per cell of *λ* = 6. **c** Simulation results displaying the number of uniquely identifiable cells over successive divisions for initial populations of 1000, 10,000, 100,000, and 1 million cells

We explored the concept of multiplet LPs, which are clusters of LPs physically bound together^25^ functioning as a single tagging unit. For instance, a 3-plet consists of three LPs that collectively serve as a unified barcode. By ensuring a minimum barcode multiplicity, multiplets reduce the occurrence of duplicate barcodes and improve the reliability of cell identification, even in large populations. Since their individual LPs (singlets) are bound together, multiplets do not separate during cell division, allowing to preserve combinatorial barcodes over successive cell divisions. This expected benefit is illustrated in Fig. 8a.

Using a revised theoretical framework, we conducted computer simulations comparing cells tagged with singlets and multiplets composed of 2-, 3-, and 4-plets. All cases assumed an identical initial average number of LPs per cell (*λ* = 6) (Fig. 8b). The parameters simulated typical experimental conditions, including the stochastic distribution of LPs among daughter cells during division. The results, shown in Fig. 8c, illustrate the number of uniquely tagged cells (for an error tolerance ${\varepsilon }_{0}$ of 1%) in samples with initial cell populations (*N*) ranging from 1000 to 1 million as they undergo multiple cycles of cell division. The findings highlight a clear advantage for multiplet tagging in maintaining a stable population of identifiable cells over successive divisions. Singlet-tagged cells exhibited a rapid decline in unique identifiers as divisions progressed. In contrast, 2-plets outperformed singlets and were suitable for small populations (*N* = 1k), while 3-plets and 4-plets were strongly preferable for larger populations, maintaining a steady number of identifiable cells. Interestingly, the simulations showed an initial increase in identifiable cells as divisions progressed. This phenomenon occurs when cells initially tagged with multiple multiplets produce daughter cells with distinct barcodes due to the particles’ distribution between progeny. Overall, these theoretical results suggest that multiplet-based tagging offers a robust and scalable strategy for maintaining unique cell identifiers over time, particularly in proliferative cell populations.

### Biocompatibility of LP tagging

**Fig. 9:**
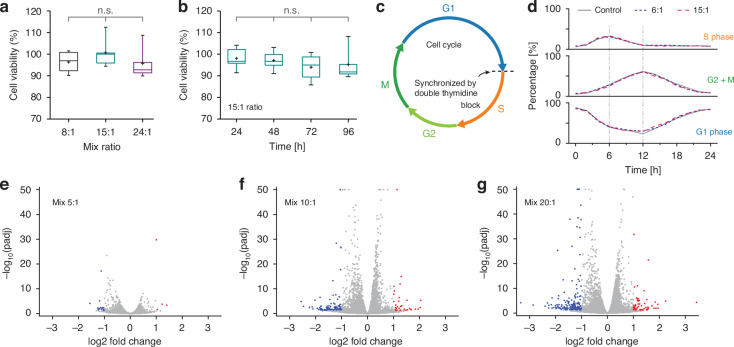
Biological effects of LP tagging on HeLa cells. **a** Cell viability after 24 h incubation with three different LP-to-cell mixing ratios, normalized to untagged control, assessed by CCK-8 assay. **b** Long-term viability of HeLa cells tagged at a 15:1 ratio. A two-way analysis of variance (ANOVA) was used for statistical analysis [*F*(2,22) = 16.47] (n.s. not significant). **c** Schematic representation of the cell cycle. **d** Percentage of cells in each cell cycle phase, evaluated at 2-h intervals for 24 h after thymidine block release. Curves for untagged cells (gray solid lines), 6:1 tagged cells (blue dotted lines), and 15:1 tagged cells (red dashed lines) are shown, indicating minimal impact on cell cycle progression. **e**–**g** Differential expression of mRNA transcripts in HeLa cells 24 h post-tagging at mixing ratios of 5:1, 10:1, and 20:1, respectively, compared to untagged controls (two samples per condition). Differentially expressed genes (log2 fold change $|{\rm{FC}}|$ > 2, adjusted *p*-value < 0.05) are marked in blue (up-regulated) or red (down-regulated)

Given the necessity of utilizing multiple LPs for large-scale combinatorial barcoding, we evaluated the potential cytotoxicity associated with our barcoding methods. Our approach is chemically and geometrically similar to magnetic beads widely used for cell separation^40^. Our previous study^11^ has shown minimal impact of antibody-coated LP tagging on viability and expression of surface markers in PBMCs. In this research, we focused on assessing the effects of PEI-LPs on viability, cell cycle progressions, and gene expression in HeLa cells. The viability of tagged versus untagged HeLa cells was measured using a Cell Counting Kit-8 (CCK-8) assay. After 24 h of incubation, no significant differences in cell viability were observed for initial LP-to-cell mixing ratios of up to 24:1 (Fig. 9a). Tagging at a 15:1 ratio did not impact cell viability over 4 days in culture (Fig. 9b). Similar results were obtained for fibroblast L929 cells (Supplementary Fig. S13). Cell cycle progression, a critical aspect of cell physiology, can indicate cytotoxicity if disrupted. To explore potential impacts, we synchronized HeLa cells using a double thymidine block^41^, effectively halting the cell cycle at the G1/S boundary (Fig. 9c). Upon thymidine removal, the cell cycle resumed, advancing through the S and G2/M phases. We monitored the cell phase distribution every 2 h for 24 h post-thymidine release, using DNA fluorescence staining to determine cell cycle phases. Tagged and untagged cell samples exhibited nearly identical progression patterns (Fig. 9d), with S phase peaks at 6 h G2/M phase peaks at 12 h. This result demonstrates the minimal effect of LP tagging on cell cycle dynamics.

We further investigated gene expression changes 24 h post-tagging via bulk mRNA sequencing, comparing tagged HeLa cells against untagged controls. Volcano plots for groups prepared with three different mixing ratios—5:1 (*λ* ≈ 3), 10:1 (*λ* ≈ 5), and 20:1 (*λ* ≈ 7–8)—are presented in Fig. 9e–g. Among a total analysis of about 15,000 genes, differential expression analysis showed 15 genes were downregulated (blue dots) and 4 genes upregulated (red dots) for the 5:1 ratio. These numbers increased to 87 downregulated and 32 upregulated genes for the 10:1 samples and to 157 downregulated and 56 upregulated genes for the 20:1 samples, indicating a dose-dependent effect on gene expression. A complete list of the differentially expressed genes can be found in the Supplementary Tables 1–3. The data indicates that, while low concentrations have minimal effects, increasing LPs tagging may lead to subtle changes on cells transcriptomic profiles. Compared to a previous study involving GaAs microparticles of similar sizes^42^, the number of differentially expressed genes is more than an order of magnitude lower in our case, suggesting that the protective silica coating helps reducing the cytotoxicity effects due to leaking of heavy metal ions from the active semiconductor material.

## Discussion

In our study, we have described a combinatorial method for generating optically readable barcodes to identify cells, tagging them with multiple LPs that emit unique sub-nanometer emission lines. Traditional barcoding techniques typically depend on discrete elements, such as A, C, G, and T bases in DNA barcoding^43^, or categorize signals into discrete levels, like in fluorescence-barcoded multiplexed beads^19^. Although it is possible to produce LPs with discrete emission lines^25,44^, for example, by e-beam lithography, large-scale production with current fabrication methods poses challenges. The LPs in this study feature continuously varying diameters, creating a spectrum of continuous emission energies over 300 meV. Barcodes are identified by calculating the distance between barcode measurements, with a theoretical framework developed to find the optimal threshold distance ($\delta$) for barcode matching taking experimental noise into consideration. This allows to determine the minimal error rate achievable for tagging a specific number of cells with defined multiplicity. This adaptable model accommodates LPs across different spectral regions, including cases where barcode lines exhibit correlations, like dual-mode LPs. Validation against experimentally measured duplicate rates (Fig. 6a) demonstrates its accuracy for multiplicities up to four, even when dual-mode LPs are considered. The model facilitates extrapolation to larger cell numbers or higher multiplicities, scenarios that are impractical to verify experimentally due to the vast number of cells required. Moreover, it allows for realistic modeling of cell pools with stochastic multiplicity distributions, reflecting the natural variability of the tagging process.

The framework offers a valuable tool for optimizing tagging parameters to meet specific experimental goals. For example, our findings indicate that with a total error rate below 1%, up to 260,000 cells can be distinctly tagged with three LPs per cell, while up to 40 million cells can be tagged with four LPs per cell (Fig. 6b). More stringent requirements, such as those for rare-cell detection in which even minimal duplication compromises data integrity, can also be accommodated. The current approach identifies barcode matches using a defined threshold for pairwise line distances. Future improvements could incorporate advanced algorithms, introducing confidence metrics to assign probabilities to matches based on actual inter-line distances. Addressing measurement discrepancies, such as those arising from varying number of detected lines, will further enhance accuracy and reliability for high-precision applications.

Long-term experiments require stable spectral barcodes, but environmental factors such as semiconductor degradation, oxidation, temperature changes, particle-particle interactions, and variations in the local refractive index can cause undesirable spectral shifts. Protective silica coatings of sufficient thickness, combined with temperature-effect calibration, mitigate these influences. Residual environmental effects can be integrated into the theoretical framework as noise contributions for realistic modeling. Enhancing LPs with omnidirectional emission^39^ will improve detection robustness, further stabilizing LP performance.

PEI-LPs, which utilizes electrostatic interactions with negatively charged cell membranes, demonstrated high tagging efficacy for adherent cells. For HeLa cells, mixing ratios of 5–8 LPs per cell effectively tagged the majority of cells with at least three LPs. In contrast, antibody-coated LPs were better suited for blood cells, such as lymphocytes, which resist internalization and depend on strong membrane binding. While antibody-mediated tagging offers high flexibility, applications requiring uniform tagging efficiency across heterogeneous cell populations, such as immune activation studies, may be biased by differential efficiencies. Optimization of antibody mixes and normalization during data analysis are essential for reliable conclusions.

The stochastic nature of LP tagging inherently leads to under-tagged cells, typically resulting in duplicate barcodes. While these duplicates can be excluded to maintain low error rates, this often necessitates discarding a significant proportion of cells (Fig. 6c). LP tagging also faces challenges in long-term studies, where extracellular uptake, LP loss, intercellular exchange, and division-induced dilution compromise barcode integrity. Miniaturization of LPs^45^ and increasing the tagging ratio^46^ partially mitigate these issues but do not eliminate variability in multiplicities or the dilution of barcodes during cell proliferation. Furthermore, applications like imaging require accurate identification of cell boundaries, a task complicated in 3D tissues by scattering and optical complexity. For these applications, multiplet LPs address many limitations of singlets, offering stable and unique barcodes across successive cell divisions. Multiplets minimize duplication errors and obviate the need for precise cell contour delineation, making them ideal for 3D imaging. Simulations demonstrate that 3-plets and 4-plets maintain a stable population of uniquely identifiable cells over time, even in large cell populations exceeding 1 million cells.

The ability to uniquely tag and identify individual cells optically on large scales opens new avenues for multi-dimensional single-cell analyses^18^, fostering a deeper understanding of individual cell states and behaviors. Beyond cell tagging, the combinatorial barcoding technology based on LPs has broader applications, enabling the tagging of microbeads for assays^19,20^ and screening merchandise with unique identifiers for tracking and anti-counterfeiting^47^.

## Materials and methods

### Laser particle fabrication

Laser Particles (LPs) were fabricated using an optical lithography method. Epitaxial structures of InGaAsP active layers grown on InP substrates were purchased from Seen Semiconductors Ltd. A typical wafer structure consists of 6 active InGaAsP layers with emission between 0.825 and 1.075 eV (equally spaced by 50 meV) and thicknesses between 292 nm and 223 nm. The active layers are separated by InP sacrificial layers of 340 nm, and capped by a protective InP layer of 500 nm. The wafers were coated with a 2 μm thick layer of SU8-2002 (Kayaku AM), followed by a pre-exposure bake (1 min at 65 °C, 2 min at 90 °C, 1 min at 65 °C). The wafers were then exposed on a mask aligner (SUSS MicroTec MA6) at a dose of 400 mJ/cm^2^ through a chrome mask containing 1.6–1.8 μm holes, followed by a post-exposure bake (1 min at 65 °C, 2 min at 90 °C, 1 min at 65 °C). The lithographic pattern was developed by immersion for 1 min in SU-8 Developer (Kayaku AM), followed by rinsing with isopropyl alcohol. The pattern was hardened with a hard-bake at 190 °C for 10 min, followed by oxygen plasma treatment (3 min, 30 sccm, 100 W). The wafers were then etched with an Ar-Cl_2_-based recipe for 20 min at 180 °C via inductively coupled plasma reactive ion etching (Oxford PlasmaPro 100 Cobra 300). Finally, the resist mask was removed via a 15 min O_2_-CF_4_ plasma etching followed by immersion in H_2_SO_4_:H_2_O bath for 30 s and washing in de-ionized water. After fabrication, LPs were transferred and coated with a protective SiO_2_ layer following a modified Stöber method, as reported before [1–2].

### Laser particle functionalization

#### PEI-LPs

Chloropropyltriethoxysilane (CPTES) coating was first performed on silica-coated LPs to introduce rich amino groups on the surface. Briefly, 18 μl of the 10% CPTES-EtOH solution (v/v) and 200 μl pure water were added into LPs solution successively. After short sonication, the LP mixture was placed on a thermomixer overnight at 70 °C and 1000 rpm to complete the reaction. The mixture was then centrifuged (4000 rcf, 8 min), followed by aspiration of the supernatant and addition of fresh EtOH (2 ml). After another brief sonication the solution was again centrifuged to remove the supernatant, and this washing cycle was repeated for at least two more times. After the final washing, we removed the supernatant EtOH and resuspended LPs in 2 ml pure H_2_O. For PEI coating, 200 μl 10% PEI (~1800 Da) water solution (v/v) was added into the LPs-CPTES and sonicated for 5 h at 80 kHz. The sample was then clean by three cycles of centrifugation/resuspension in EtOH. After the final wash, the PEI-LPs ware resuspended into an appropriate amount of pure H_2_O to bring the final concentration of LPs to 1 M/10 μl.

#### Biotin-LPs

For the functionalization of Laser Particles (LPs) with biotin, the silica-coated LPs were first resuspended in 1 mL of 95% EtOH. Separately, 20 mg of Biotin-PEG2000-Silane was dissolved in 1 mL of 95% EtOH. The Biotin-PEG2000-Silane solution was then added to the LP suspension, and the mixture was incubated overnight at 65 °C to ensure complete reaction. Afterwards, the LPs were centrifuged at 2000 × *g* for 8 min, and the supernatant was removed. The LPs were washed with 4 mL of clean 95% EtOH, briefly sonicated, and centrifuged again at 2000 × *g* for 8 min. This washing step with 95% EtOH was repeated, followed by an additional wash with 4 mL of deionized (DI) water. After the final wash, the biotin-functionalized LPs were resuspended in 1 mL of DI water.

### Cell culture

Most cell cultures used, including fibroblasts (L929 human cells), adenocarcinoma cells (HeLa human cancer cells, 4T1 mouse triple-negative breast cancer cells and MCF-7 human breast cancer cells), macrophage cells (RAW 264.7 mouse cells) and Jurkat T lymphocyte were purchased from American Type Culture Collection (ATCC). Mouse cortical stem cells were obtained from R&D Systems™ (Catalog Number NSC002). Mouse splenocytes were obtained from ScienCell^TM^ (Catalog Number #M5540), human KOPN-8 cells were Deutsche Sammlung von Mikroorganismen und Zelkuturen GmbH (Braunschweig, Germany), while suspension human PBMC cells were from ZenBio (lot #PBMC042022A).

All cells were cultured and maintained following standard protocols from the supplier and incubated at 37 °C in a humidified atmosphere of 5% CO_2_ and 95% air. Fibroblast cells, macrophage cells, and most adenocarcinoma carcinoma cells (Hela, 4T1, and MDA-MB-231) were cultured in DMEM essential cell media supplemented with fetal bovine serum (10%, v/v) and penicillin/ streptomycin (1%, v/v). MCF-7 cells and Jurkat T-cells were cultured in RPMI-1640 medium supplemented with FBS (10%, v/v), and penicillin/ streptomycin (1%, v/v). Cortical stem cells were cultured in DMEM media, with 2% N21-Max supplement and 1% penicillin/ streptomycin, with daily additions of fibroblast- and epidermal-growth (FGF basic and EGF, 20 ng/mL). Pre-coating the culture plates with poly-L-ornithine and fibronectin was needed for cortical stem cell cultures. Primary mouse splenocytes and their isolated T cells were cultured and stimulated in RPMI-1640 supplemented with FBS (10%, v/v), P/S (1%, v/v), sodium pyruvate (1%, v/v), 1× non-essential amino acids, 10 mM HEPES buffer, 1 × 2-mercaptoethanol, recombinant mouse IL-2 (carrier-free) (10,000 U/mL), recombinant mouse IL-7 (carrier-free) (5 ng/mL), and recombinant mouse IL-15 (carrier-free) (10 ng/mL).

For primary immune cell work, cryopreserved human PBMCs were quickly thawed on the same day as tagging using a pre-warmed thawing medium (RPMI-1640 medium supplemented with FBS (20%, v/v), and penicillin-streptomycin (1%, v/v)). After being washed twice with thawing medium, the PBMCs were resuspended and incubated in 1 mL of DNase solution for 15 min (0.1 mg bovine pancreatic DNase I in 1 mL of RPMI-1640 medium) at 4 °C. The cells were then washed with a wash buffer (10% FBS, 1× Pluronic F-68, 2 mM EDTA, and 10 mM HEPES in PBS) prior to LP tagging. For isolated T cell work, CD3 + T cells were isolated following the EasySep^TM^ human T cell isolation kit protocol (STEMCELL). Primary mouse splenocytes were harvested from BALB/c mice. They were stimulated with phytohemagglutinin for 48 h, then stimulated with phorbol 12-myristate 13-acetate (PMA) overnight. Prior to tagging, the splenocytes’ CD3 + T cells were isolated following the EasySepTM mouse T cell isolation kit protocol (STEMCELL). Following standard protocol, human KOPN-8 cells were thawed in RPMI-1640 medium supplemented with 20% fetal bovine serum and cultured in RPMI-1640 medium with 10% fetal bovine serum and 1% penicillin/streptomycin.

### Tagging cells with PEI-LPs

Once confluent, adherent cell cultures were passaged by washing with PBS and then detached by incubating in TrypLE Express (Life Technologies) at 37 °C for 5 min, washed with complete media, centrifuged (400 g for 5 min) to remove the supernatant and resuspend in fresh media. For each tagging experiment, 100 k cells were taken into a 1.5 mL tube and diluted to reach a final concentration of 100k cells/100 µl. In the meantime, PEI-LPs in sterile water were briefly sonicated to resuspend and fully disperse them. An appropriate volume of LPs solution (based on the desired final tagging ratio) was then mixed to PBS at a concentration of 500 k LPs/100 μl PBS. The LPs solution was mixed to the cell tube in 5 separate equal additions, with 1/5 of the volume added every 10 min. The cell tube was kept on a thermomixer at 37 °C, 800 rpm between additions. After the final addition, the cell-LPs solution was kept on the mixer for another 30 min. Finally, the cells were centrifuged (400 g for 5 min), resuspended in fresh complete cell media, gently pipetted/mixed ten times, and seeded on culture plates at the desired concentration.

### Tagging cells with Ab-LPs

PBMC samples were stained with 1.0 µg anti-CD3-biotin or 1.0 µg anti-CD19-biotin each for 15 min at 4 °C in wash buffer, washed, then incubated with 10 µg of purified streptavidin each for 25 min at 4 °C in wash buffer and washed. In 1 mL per 500k cells, samples were tagged with biotin-coated LPs by mixing the LP solution with the sample at a ratio of 10 LPs to 1 cell, and promptly compensating the with an appropriate amount of 10× PBS to offset the deionized water of the LP solution. Samples were then mixed on a thermomixer at 650 rpm while at 4 °C for 5 min, then centrifuged at 200 g for 5 min. Mixing and centrifugation was repeated twice.

Isolated human T cells were tagged as above using anti-CD3-biotin. KOPN-8 cells were tagged as above but using anti-β2M-biotin. Mouse splenocyte-derived T cells were tagged as above but using anti-H-2Kd-biotin and anti-CD45-biotin.

### Assessment of LPs tagging

Images of PEI-LPs tagged adherent cell cultures were taken after 24 h incubation (upon tagging at a 5:1 LP to cell ratio), following fixation in 4% paraformaldehyde (PFA). Images were taken using a brightfield inverted microscope (Olympus IX83). Tagging efficiency estimations for HeLa and MCF7 cells were performed by culturing the tagged samples at different initial tagging ratios and culturing for different time intervals, as described in the main text. After the appropriate culture time, the PEI-LPs-tagged cells were fixed and several images per sample were taken. The number of LPs in each cell was counted manually from the collected images for at least 100 cells per sample.

High-resolution 3D images of LP localization in the cytoplasm were obtained using a confocal fluorescence microscope (Olympus FV3000). LP localization was obtained by staining them with an Alexa Fluor™ 488 TFP ester (Life Technologies) by direct conjugation with the anime groups on the surface of LPs-PEI through typical amine-TFP crosslinker chemistry. Then, Hela cells were tagged by PEI-LPs/AF488 and fixed by 4% PFA after incubation at 6 h and 24 h. The cell nuclei were stained with (4′,6-diamidino-2-phenylindole dihydrochloride) (DAPI), while membranes were stained by CellMask™ deep red plasma membrane staining kit (Life Technologies) following the manufacturer’s protocol.

To assess the tagging efficiency of suspension cells (PBMCs, KOPN-8, mouse splenocytes), the LASE flow cytometer (see below) was used to record the number of emission lines for each cell.

### Measurements of LPs barcodes

Barcode measurements of tagged cells were performed using two different instruments capable of measuring the laser emission spectra of LPs: a confocal microscope (Olympus FV3000) and a flow cytometer (Beckman Coulter CytoFLEX). Both instruments were modified by adding a 1064 nm, 10 ns pulsed laser to pump the LPs and an IR-spectrometer coupled to a SWIR camera (Sensor Unlimited GL2048L) to measure their emission spectra. For threshold measurements, the pump power on the confocal microscope was controlled with an acousto-optic modulator (QuantaTech). For long-term measurements, a stage-top incubator was used to maintain a constant temperature of 37 °C and a CO_2_ concentration of 5%. More information on the instruments can be found in our previous publications^11,25^.

Both instruments return a list of spectra, either one per pixel (in the case of the microscope) or one per event (in the case of the flow cytometer). These spectra are analyzed in post-processing with a custom pipeline developed in Python. First, a peak-finding algorithm is applied to each spectrum (*S*_*i*_) to identify the different emission lines. Each peak is then fitted with a gaussian function to better estimate its central energy (*E*_*i,j*_, *j* = 1…*M*_*i*_, where *M*_*i*_ is the number of peaks in the spectrum). For the flow cytometry data, each cell is assigned a single spectrum, and the cell barcode is just formed by the spectral position (*E*_*i,1*_*,…,E*_*i,M*_) of the emission lines of that spectrum.

In the case of imaging data, multiple spectra can be assigned to each LP (based on scanning pixel size and LP emission properties). A clustering algorithm is applied to the data extracted from the spectra to identify spatially adjacent peaks with similar energy, with each resulting cluster corresponding to the emission of a single LP. The average energy of the peaks in the cluster is taken as the emission energy of that LP. The collection of all energies of the LPs inside an individual cell is then taken as its barcode. In the current work, LP assignment to cells in imaging data was performed manually.

### HeLa cell matching

To validate barcoding and matching across two cycles of flow cytometry, HeLa cells were tagged with PEI-LPs at a mixing ratio of 5:1, as described previously, and incubated for 24 h. Subsequently, the cells were stained with CellTracker Green (Invitrogen) at a concentration of 1 µM for 15 min, following the manufacturer’s instructions. After staining, the cells were rinsed, treated with trypsin, detached from the culture dish, and resuspended in about 200 µL of wash buffer (10% FBS, 1× Pluronic F-68, 2 mM EDTA, and 10 mM HEPES in PBS). A first measurement was performed using the LASE flow cytometer. Following this, the cells were collected in a 5 mL tube preloaded with 500 µL of wash buffer, centrifuged at 300 g for 7 min, and the supernatant was removed. The cells were then resuspended in 200 µL of wash buffer and measured a second time with the flow cytometer.

To match cells between the two measurement cycles (*C*_*1*_ and *C*_*2*_), we used a custom algorithm that scores possible matches based on their likelihood. For two barcodes (${{\boldsymbol{E}}}_{1}$ and ${{\boldsymbol{E}}}_{2}$), the algorithm assigns a score $s({{\boldsymbol{E}}}_{1},{{\boldsymbol{E}}}_{2})$ based on the energy distance between their lines. For barcodes with differing multiplicities, the algorithm considers the subsets of closely aligned line pairs (within 1.5 meV) and applies penalties for unmatched lines. Two sets of scores were calculated: “cross-matches” (*S*_*cross*_) between *C*_*1*_ and *C*_*2*_, and the union of “self-matches” (*S*_*self*_) between each dataset (*C*_*1*_*-C*_*1*_, *S*_*11*_ and *C*_*2*_-*C*_*2*_, *S*_*22*_), excluding self-comparisons. The *S*_*cross*_ set contains a mix of correct matches and incorrect matches caused by nearby barcodes, while the *S*_*self*_ set contains only incorrect matches. By comparing the score distributions for *S*_*cross*_ and *S*_*self*_, the *S*_*cross*_ set reveals a subpopulation with high scores corresponding to correct matches, which are absent in *S*_*self*_. The scoring function was optimized to maximize the size of the subpopulation of correct matches in *S*_*cross*_. Finally, all matches from *S*_*cross*_ with scores exceeding a threshold were selected, ensuring a correlation of >98% in fluorescence signals.

### Biocompatibility assessment of PEI-LPs tagged cells

The in vitro cytotoxicity of PEI-LPs was quantitatively assessed using CCK-8 (ApexBio) assay for L929 and Hela cells. Specifically, three groups of cells were tagged separately with different mixing ratios of PEI-LP (LP/cell = 8, 15, 24). After co-incubation for 24 h in a 96-well plate ( ~ 3k cells/well), CCK-8 reagents were added following manufacturer protocols (*n* = 6). Untagged cells at 24 h were used as a control group. Absorbance measurements were taken using a spectrophotometer (Epoch 2, Biotek Instruments). For time-dependent cytotoxicity, cells (~ 3k cells/well) tagged at an initial ratio of 15:1 (LP:cell) were co-incubated 24, 48, 72, and 96 h in separate wells, and the cell viability was evaluated with the CCK-8 method, with untagged control cells taken at the same time points (*n* = 6).

The cell cycle progression of LPs-tagged cells was determined through the cell synchronization method using a double thymidine block. HeLa cells were tagged with PEI-LPs at initial ratios of 6:1 and 15:1 (LP:HeLa) and then plated into 96-well glass-bottom microplates at a density of about 3000 cells/well. Untagged HeLa cells were used as a control group. After overnight incubation for LPs-tagged HeLa cells (37 °C, 5% CO_2_), an appropriate thymidine stock solution (100 mM) was added to each well to obtain a final concentration of 2 mM. The plates were returned to the incubator and incubated for 20 h to arrest most cells in the G1/S stage. Afterwards, the cells were washed three times with pre-warmed PBS buffer and fresh media was added. After 8 h of further incubation, a second thymidine addition (2 mM final concentration) was performed for 16 h. The cells were then released by washing them three times with pre-warmed PBS buffer and replacing with fresh media. The plates were returned to the incubator, and four wells for each group were fixed every 2 h over 24 h. At the end, all fixed cells were stained with Hoechst 33342 (10 µM) for 15 min, followed by two washes with 200 µl of DPBS. A few fluorescence images of each well were recorded using a confocal microscope (Olympus FV3000) with a 10× objective. The fluorescence intensity of the nuclei of each cell was quantitatively calculated using ImageJ and then presented as a cell number-fluorescence intensity distribution plot, from which the proportions of cells in different stages (G1, S, and G2) were obtained at different time points for the three groups (*n* = 6).

### Bulk RNA-seq samples preparation

Hela cells tagged with PEI-LPs were used for bulk RNA-seq experiments. Fresh HeLa cells from ATCC (CCL-2) were first thawed and cultured following standard protocols for two generations. Cells were then harvested and tagged with PEI-LPs (as described above) in four different groups: untagged, 5:1, 10:1, and 20:1 (LPs:cell). Two replicates for each group were made, with 80 k cells in each replicate. All cell samples were prepared in parallel and using the same batch of PEI-LPs. After 24 h of incubation, HeLa cells were harvested, suspended in PBS buffer, and used for RNA extraction using the Direct-zol™ RNA Miniprep Plus kit (Zymo Research). Three times the volume of TRI Reagent was added to each sample and mixed thoroughly to lyse cells and then transferred to RNA purification through primary RNA harvesting by Zymo-Spin™ IIICG Column, removing DNA by DNA DNase I treatment and several cycles of washing by RNA Wash Buffer. The RNA obtained in this way was redispersed in DNase/RNase-free water and immediately moved to a −80 °C refrigerator for storage. Quality control test of all RNA samples was conducted with the Agilent 2200 TapeStation system using RNA ScreenTape, RNA ScreenTape sample buffer, and RNA ScreenTape ladder (Agilent). The samples were then shipped overnight in dry-ice for sequencing at an external company (MedGenome Inc.).

### Bulk RNA-seq data analysis

The quality of reads was assessed using FastQC (v0.11.8). Key parameters evaluated included base quality score distribution, sequence quality score distribution, average base content per read, GC content distribution, detection of PCR amplification issues, and over-represented sequences. Adapter sequences were trimmed using fastq-mcf (v1.05) and cutadapt (v2.5). Sequences with low quality were excluded from further analysis. To eliminate unwanted sequences, Bowtie2 (v2.5.1) was employed. Non-polyA tailed RNAs, mitochondrial genome sequences, ribosomal RNAs, transfer RNAs, and remaining adapter sequences were removed to ensure clean RNA-Seq data. The cleaned reads were aligned to the reference human genome (GRCh37/hg19) using STAR (v2.7.3a). This aligner is known for its high speed and accuracy, employing a two-pass mapping strategy to enhance detection of novel splice junctions.

Gene expression levels were quantified using HTSeq (v0.11.2) to count reads mapping to gene exons, followed by normalization with DESeq2. Additionally, expression values were reported in FPKM units using cufflinks (v2.2.1). Quality control of RNA-Seq data was further validated with RNA-SeQC (v1.1.8), RSeQC (v3.0.1), and MultiQC (v1.7). Differential expression analysis was performed with the R Bioconductor DESeq2 package.

### Data analyses and numerical simulations

All data analyses and numerical simulations were performed with custom scripts written in Python, using the numpy, scipy, matplotlib and scikit-learn libraries.

## Supplementary information

Supplementary Information

## Data Availability

The data supporting the findings of this study are available from the corresponding author upon reasonable request. The sequencing data is available from the NIH Sequence Read Archive (SRA) under accession number PRJNA1230263.

## Code availability

The code for the analyses described in this paper is available from the corresponding author upon reasonable request.
