# Supplementary material for: Large-scale combinatorial optical barcoding of cells with laser particles
Source: Light Sci Appl. 2025 Apr 1;14:148. doi: 10.1038/s41377-025-01809-x (PMC11962087; doi:10.1038/s41377-025-01809-x)
Supplement: Supplementary file 1 — Supplementary Information [file 41377_2025_1809_MOESM1_ESM.pdf]

Supplementary Information

Large-Scale Combinatorial Optical Barcoding of Cells with Laser Particles

Nicola Martino[1,2,†], Hao Yan[1,3,†], Geoffrey Abbott[4], Marissa Fahlberg[4], Sarah Forward[4], Kwon-Hyeon Kim[1], Yue Wu[1,2], Han Zhu[4], Sheldon J.J. Kwok[4], Seok-Hyun Yun[1,2,5,*]

[1] Harvard Medical School and Wellman Center for Photomedicine, Massachusetts General Hospital, 65 Landsdowne St., Cambridge, MA 02139, USA.

[2] Broad Institute of MIT and Harvard, 415 Main St. Cambridge, MA 02142, USA.

[3] Current address: Tsinghua Shenzhen International Graduate School, Tsinghua University, Shenzhen 518055, China.

[4] LASE Innovation Inc., 335 Bear Hill Rd. Waltham, MA 02451, USA.

[5] Harvard-MIT Health Sciences and Technology, Massachusetts Institute of Technology, 77 Massachusetts Avenue, Cambridge, MA 02139, USA.

[†] These authors contributed equally to this work

[*] Corresponding author, syun@mgh.harvard.edu

Supplementary Figure 1

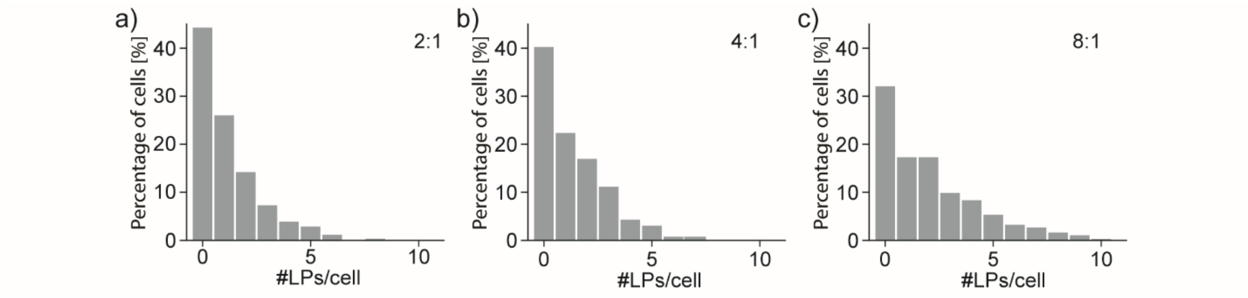

Figure S1. Tagging statistics for MDCK-II cells incubated 24 h with LPs (silica coated) at different initial concentrations of LPs/cell: (a) 2:1, (b) 4:1, (c) 8:1. Adapted from Martino et al., *Nature Photonics* **13**, 720-727 (2019).

Supplementary Figure 2

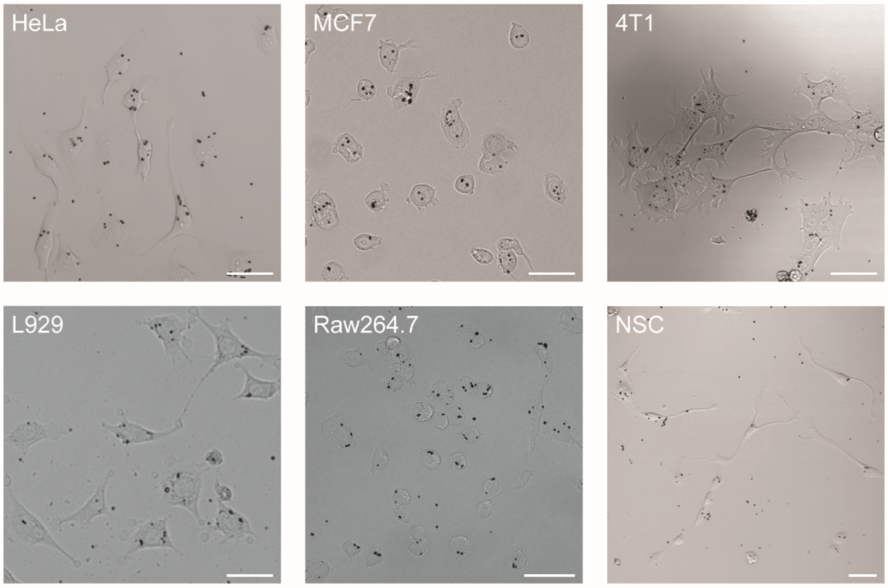

Figure S2. Brightfield images of different types of cells tagged with PEI-LPs: cancerous cell lines (HeLa, MCF7, 4T1), epithelial cells (L929), macrophages (Raw 264.7), and mouse cortical neural stem cells (NSC). Scale bar 50 μm.

Supplementary Figure 3

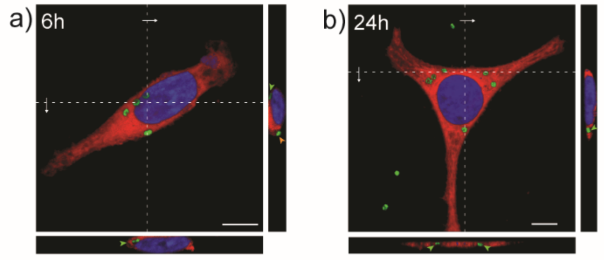

Figure S3. Confocal 3D stacks (12 µm, 200 nm steps along axial direction) of two HeLa cells fixed at 6 h (a) and 24 h (b) after co-incubation. The nucleus (blue) was stained with Hoechst 33342, the cell membrane was stained with DiD cell-labeling solution (red), and the PEI-LPs were labeled with Alexa Fluor™ 488 (AF488). Lateral panels are axial cuts along the dashed lines; small arrows identify LPs either internalized (green) or on the surface (orange). Scale bars are 10 µm.

Supplementary Figure 4

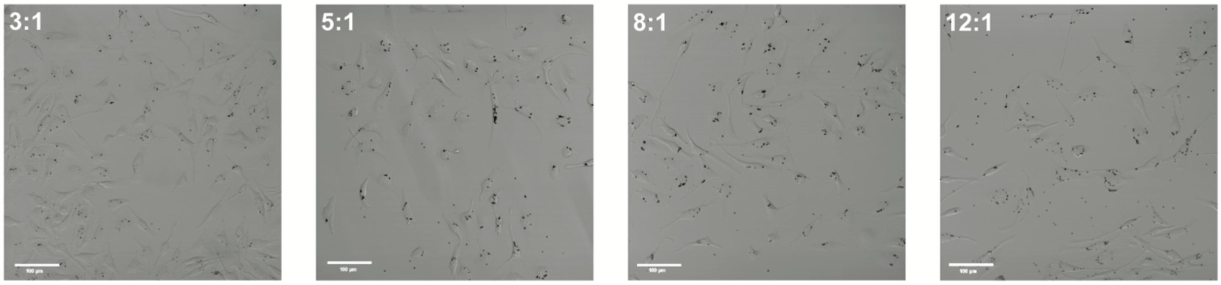

Figure S4. Representative brightfield images of HeLa cells tagged with PEI-LPs at different tagging concentrations (LPs/cell). Scale bar 100 µm.

Supplementary Figure 5

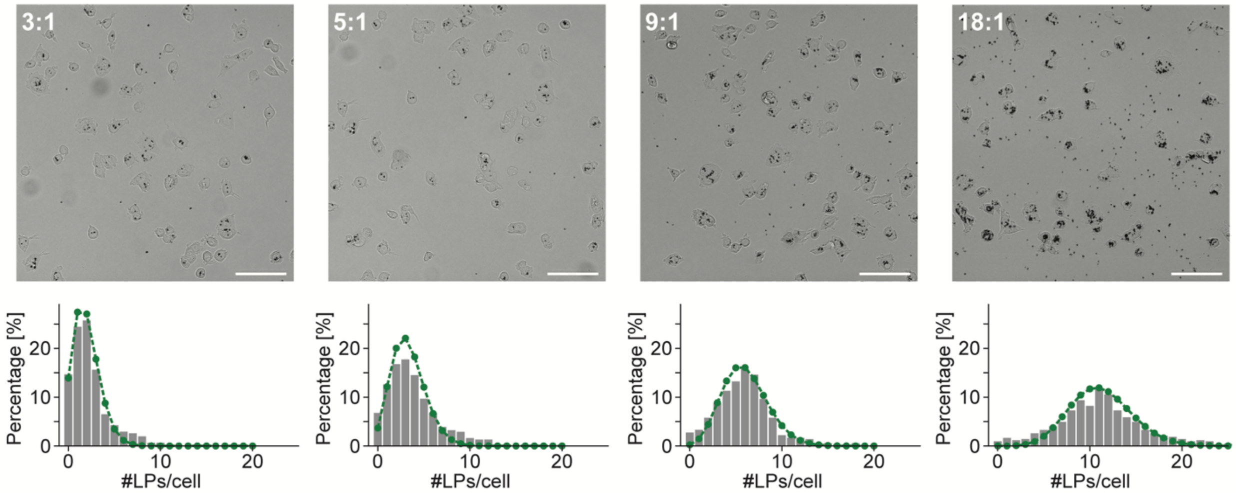

Figure S5. Representative brightfield images (top) and uptake statistics (bottom) of MCF7 cells tagged with PEI-LPs at different tagging concentrations (LPs/cell). The green dashed line is the fitted Poisson distribution. Scale bar 100 µm.

Supplementary Figure 6

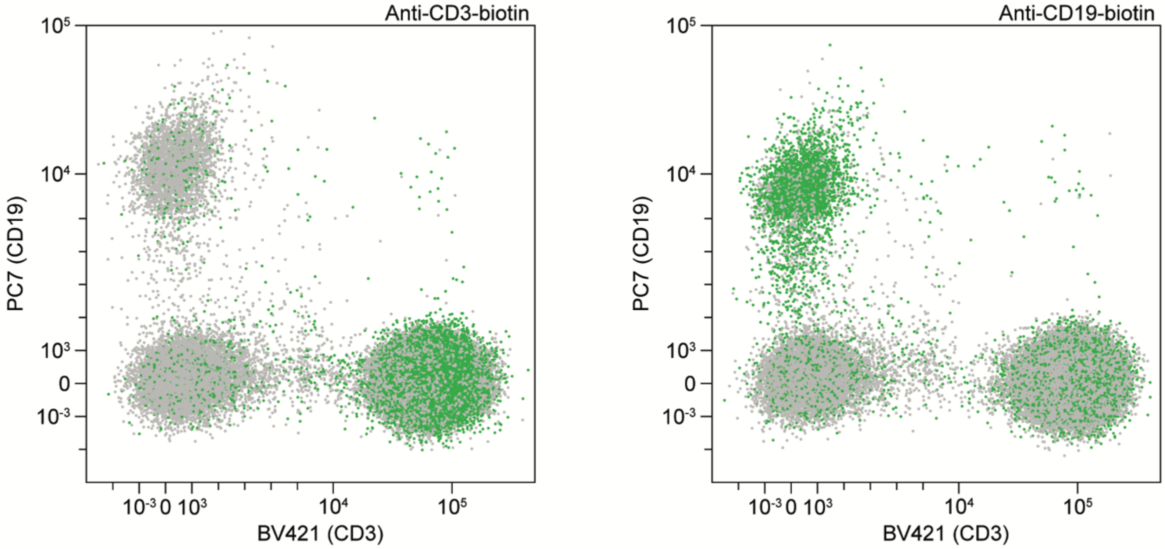

Figure S6. Targeted tagging of CD3+ T-cells (left) or CD19+ B-cells (right) in a PBMCs sample, using biotin-LPs with specific antibodies (anti-CD3-biotin and anti-CD19-biotin respectively). Grey dots represented untagged cells, while green dots represent cells tagged with at least one biotin-LP.

Supplementary Figure 7

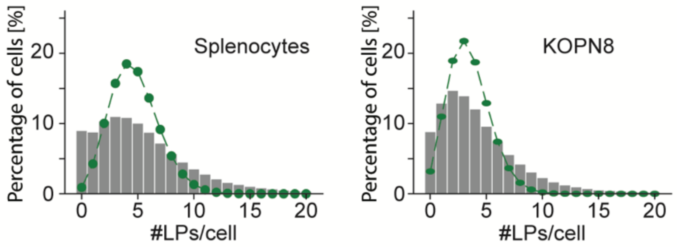

Figure S7. Uptake distributions of biotin-LPs for mouse splenocytes (left) and human leukemia KOPN8 cell line (right) using a mixture of anti-H-2Kd-biotin plus anti-CD45-biotin (splenocytes) or anti-β2M-biotin (KOPN8).

<u>**Supplementary Figure 8**</u>

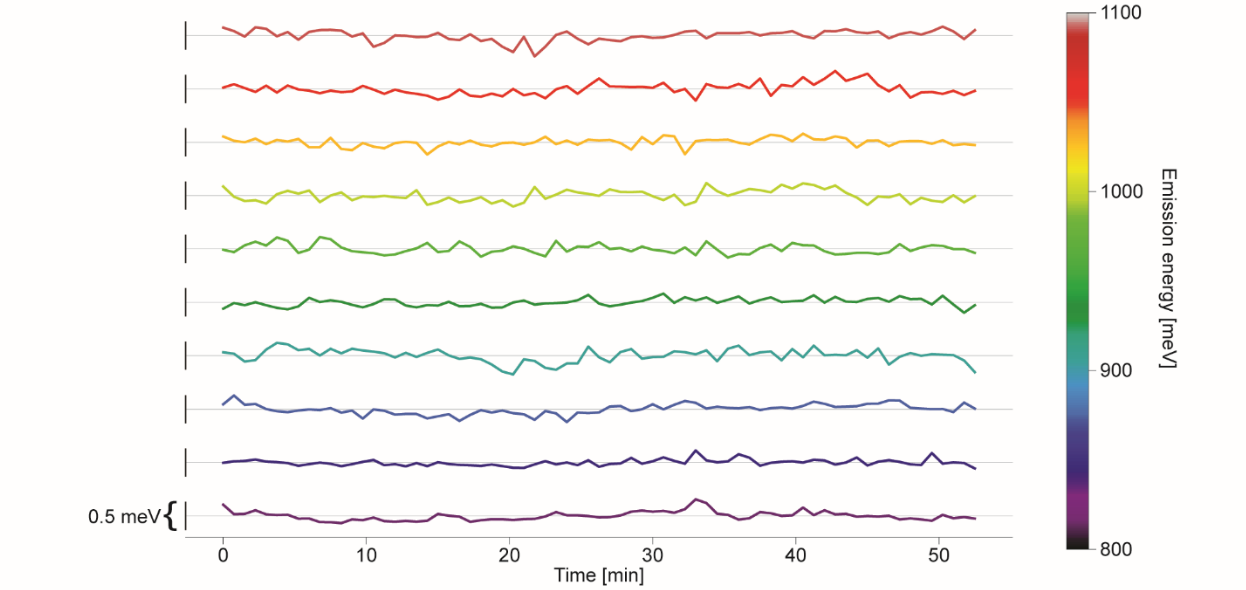

Figure S8. Representative traces of LP energy measurement over time at different central energy of LP emission (color coded as in the color-bar on the right). The LPs are internalized inside HeLa cells, and their emission measured 71 times at intervals of 45 s. The vertical bar on the left of each trace represents a 0.5 meV total shift (±0.25 meV with respect to the average energy of the full trace, horizontal solid lines).

Supplementary Figure 9

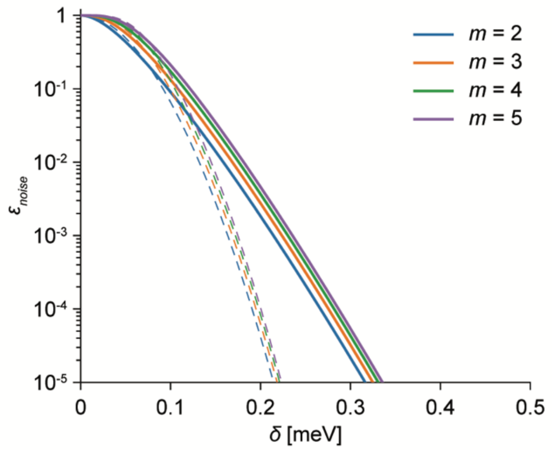

Figure S9. Noise error rate ε_{noise} using the noise probability distribution derived from experimental data, modeled as a generalized normal function with α = 0.047 meV, β = 1.28 (solid lines); the data is compared to that coming from a Gaussians pdf with σ = 0.047 meV (dashed lines).

Supplementary Figure 10

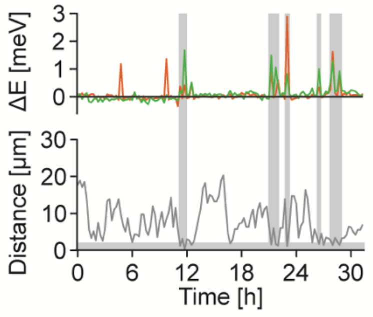

Figure S10. Emission energies of two thin-coated LPs (30 nm silica shell) within a HeLa cell (top) as their distance varies (bottom), showing large shifts up to several nm as they come into physical contact (grey shaded areas).

Supplementary Figure 11

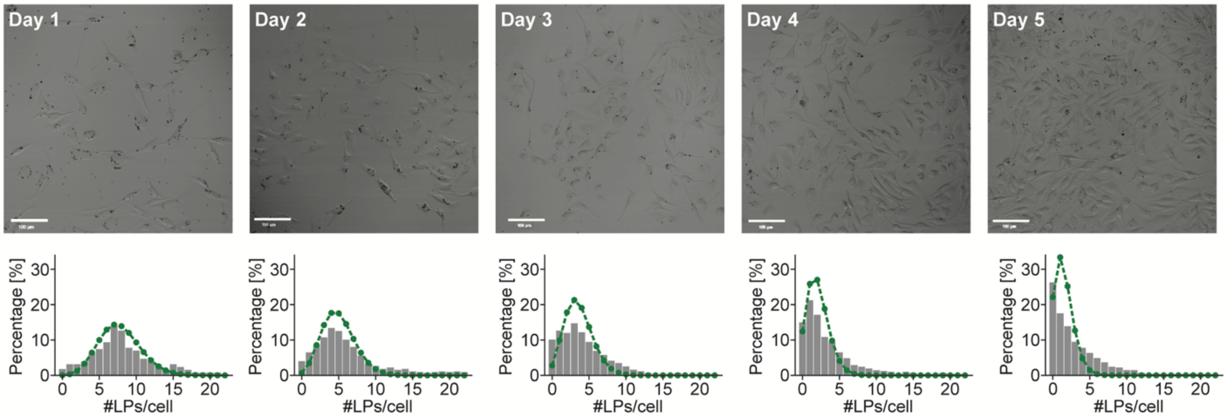

Figure S11. Representative brightfield images (top) and uptake statistics (bottom) of HeLa cells tagged with PEI-LPs at different time points after co-incubation. The green dashed line is the fitted Poisson distribution. Scale bar 100 µm.

Supplementary Figure 12

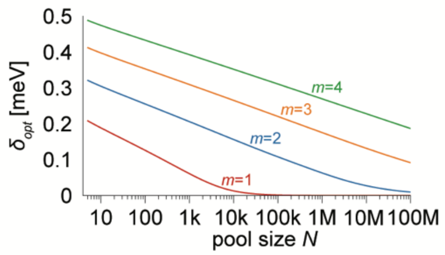

Figure S12. Optimal value of the threshold parameter δ for the experimental conditions analyzed in Figure 6 of the main text.

Supplementary Figure 13

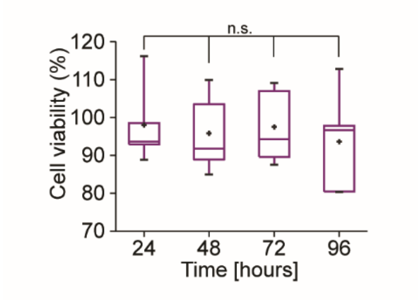

Figure S13. CCK-8 viability assay for L929 cells tagged with PEI-LPs at an initial concentration of 15:1 performed every 24 h up to 4 days after plating. Unpaired two-sided Student's t-tests were used to analyze the data (n.s., not significant).

Supplementary Note A

Let's consider LPs fabricated following a spectral distribution given by the probability density function (pdf) $g(E)$. A barcode of multiplicity m can be expressed as a sorted list of energies $(E_1, \ldots, E_m)$, where the sorting reflects the fact that the individual lines composing the barcodes are indistinguishable, and thus order does not matter. The distribution of available barcodes can be written as:

$$G_m(E_1, \ldots, E_m) = \begin{cases} m! \prod_{i=1,\ldots m} g(E_i), & \text{if } E_j > E_i \ \forall j > i \\ 0, & \text{otherwise} \end{cases}$$

Where the multiplication factor $m!$ ensures that G_m has unit integral. Given a specific barcode $\overline{\boldsymbol{E}} = (\bar{E}_1, \ldots, \bar{E}_m)$, the probability that a second barcode randomly chosen from the pdf G_m is a duplicate (*i.e.* that all their energies are within δ from each other) can be calculated as the integral of G_m in a region $\omega(\bar{\mathrm{E}})$ around $\overline{\boldsymbol{E}}$ determined by the metric used for matching:

$$p_{pair}(\overline{\boldsymbol{E}}) = \int_{\omega(\bar{\mathbf{E}})} G_m(\boldsymbol{E}) d\boldsymbol{E} \approx V_m(\delta) G_m(\overline{\boldsymbol{E}})$$

where $V_m(\delta)$ is the volume of ω and we used the approximation that G can be considered constant on the length scale of ω. In the case of the Chebyshev metric, ω is a (hyper)cube of side 2δ centered around $\overline{\boldsymbol{E}}$ with volume $V_m(\delta) = (2\delta)^m$.

If we now consider a pool of N barcodes, the probability that each specific barcode ($\overline{\boldsymbol{E}}$) is unique (*i.e.* it doesn't have any duplicate in the pool) is given by:

$$p_{unique}(\overline{\boldsymbol{E}}) = (1 - V_m(\delta) G(\overline{\boldsymbol{E}}))^{N-1}$$

The expected fraction of duplicate (non-unique) barcodes in the pool (ε) can then be estimated from the average of p_{unique} weighted by the pdf G:

$$\varepsilon_{dup} = 1 - \int G(\boldsymbol{E})(1 - V_m(\delta) G(\boldsymbol{E}))^{N-1} d\boldsymbol{E}$$

In the case of LPs following a uniform distribution in a range Δ, $g(E) = \Delta^{-1}$ and $G(E) = m!\,\Delta^{-m}$, from which we can simplify the previous expression to:

$$\varepsilon_{dup,unif} = 1 - \left(1 - m!\left(\frac{2\delta}{\Delta}\right)^m\right)^{N-1}$$

We can see that the duplicate rate for the uniform case is equivalent to that of a discrete set of barcodes by defining an effective number of barcodes $B_{eff} = \frac{1}{m!}\left(\frac{\Delta}{2\delta}\right)^m$. This value represents the ratio between $\Delta^m/m!$ (the full volume in which the barcodes are defined), and the volume occupied by an individual barcode $(2\delta)^m$.

Note that different metrics could be used; for example, using a Euclidean distance ω would become a hypersphere of radius δ.

Supplementary Note B

Let's consider a barcode with multiplicity *m* and energies $\boldsymbol{E} = (\bar{E}_1, \dots, \bar{E}_m)$. A measurement of the barcode will yield an observation $\boldsymbol{X} = (X_1, \dots, X_m)$. We call $\varphi_0(x)$ the noise distribution of the energy measurement for the individual lines of the barcode:

$$\varphi_0(x) = P(\bar{E}_i - X_i = x)$$

In general, however, the original values of the barcode lines ($\bar{E}_i$) are not known and we compare two experimental observations $\boldsymbol{X}$ and $\boldsymbol{Y}$. In this case, the noise distribution is given by:

$$\varphi(x) = P(X_i - Y_i = x) = \int \varphi_0(s)\varphi_0(s + x)ds$$

The probability that, for a single line in the barcode, the noise is within a threshold δ is given by:

$$\phi(\delta) = \int_{-\delta}^{\delta} \varphi(x)dx$$

If we use a Chebyshev metric, the probability that the two observations yield matching barcodes is equivalent to have for each of the *m* lines in the barcode a noise level within δ (*i.e.* $\phi(\delta)^m$). The error rate due to the noise (*i.e.* the fraction of not matching barcodes) is thus:

$$\varepsilon_{noise} = 1 - \phi(\delta)^m$$

Supplementary Table 1

Differentially expressed genes in 5:1 sample vs untagged control (CTRL).

gene	Mean counts (5:1)	Mean counts (CTRL)	Log2FoldChange	p_adj
CYSLTR2	159.030	468.720	-1.558	7.808E-05
NRG2	115.468	275.588	-1.256	5.661E-03
LTB4R	81.114	191.274	-1.238	3.213E-02
B3GALT4	104.042	240.365	-1.208	1.432E-02
RP11-480A16.1	111.813	257.850	-1.205	1.250E-02
CASC15	139.037	315.611	-1.183	9.974E-03
P2RY6	271.412	614.931	-1.180	7.627E-06
TMEM238	185.893	407.787	-1.134	3.633E-03
LRRC32	1282.946	2790.336	-1.121	6.798E-18
FBLN7	185.653	399.792	-1.108	1.812E-02
FAXDC2	155.576	332.119	-1.094	1.280E-02
NEAT1	9396.949	19550.193	-1.057	3.484E-03
NCCRP1	212.456	437.886	-1.044	4.948E-03
ABCA8	213.217	434.606	-1.029	2.711E-02
NRCAM	131.372	266.581	-1.021	4.348E-02
SERPINE2	12636.165	6307.254	1.002	1.733E-30
DOPEY2	404.728	196.520	1.041	2.293E-02
UBASH3B	495.442	212.184	1.223	1.742E-04
CACNB4	338.475	128.099	1.400	4.028E-04

Supplementary Table 2

Differentially expressed genes in 10:1 sample vs untagged control (CTRL).

gene	Mean counts (10:1)	Mean counts (CTRL)	Log2FoldChange	p_adj
HIST1H4A	133.079	22.598	-2.550	1.380E-05
TTLL6	104.405	18.620	-2.462	1.149E-03
CHADL	86.621	18.547	-2.237	3.612E-03
CCL8	81.160	17.218	-2.233	3.230E-03
PADI2	116.930	27.120	-2.128	1.934E-03
TENM3	77.896	17.960	-2.099	1.281E-02
ALPI	260.462	64.331	-2.024	4.357E-06
RASGRP3	136.941	36.148	-1.937	7.099E-03
CHRD	105.072	28.904	-1.881	2.471E-02
HIST2H4A	100.210	28.022	-1.838	4.393E-03
CRISPLD2	360.645	102.380	-1.826	2.848E-07
RAB11FIP4	80.264	22.892	-1.817	2.027E-02
IL17D	80.162	22.973	-1.803	1.379E-02
CALHM3	82.891	23.545	-1.798	3.815E-02
NOXA1	145.874	41.886	-1.798	1.162E-03
RP11-67L2.2	139.075	40.272	-1.783	1.007E-03
QRICH2	95.114	27.523	-1.782	9.492E-03
RP11-13K12.5	85.190	24.962	-1.777	1.363E-02
NWD1	96.810	28.639	-1.747	2.309E-02
AC087294.2	74.802	22.304	-1.731	4.197E-02
CCL7	73.569	23.391	-1.666	4.240E-02
PTPRH	79.930	25.079	-1.661	3.176E-02
MIAT	86.292	27.531	-1.660	2.801E-02
GALNT6	80.295	26.290	-1.625	4.406E-02
RP11-380J14.1	82.560	27.406	-1.601	3.248E-02
PIGZ	157.298	52.779	-1.580	3.271E-03
ARHGAP36	87.226	29.351	-1.580	4.412E-02
MMP24	126.521	42.885	-1.565	5.149E-03
PTP4A3	118.530	40.191	-1.560	8.167E-03
BMPER	179.410	61.513	-1.549	1.101E-03
FILIP1	97.279	33.527	-1.535	1.931E-02
TPT1P1	104.738	36.176	-1.532	2.294E-02
CBLN3	93.816	32.661	-1.527	2.389E-02
ADAMTS2	155.131	54.276	-1.526	6.740E-03
RP3-437C15.1	116.197	40.779	-1.521	1.580E-02
RP11-991C1.1	138.310	48.676	-1.505	4.456E-03
B3GALT4	216.308	76.895	-1.484	6.504E-04

LINC01132	133.820	48.514	-1.468	2.021E-02
PDZK1IP1	145.901	53.314	-1.453	8.116E-03
NCCRP1	394.080	144.758	-1.448	2.103E-06
LYPD5	99.944	37.086	-1.429	2.582E-02
UGT3A1	227.701	85.447	-1.423	1.486E-03
IGFBP5	284.910	107.869	-1.401	9.012E-05
C11orf35	116.799	44.331	-1.398	2.309E-02
RP11-359E3.4	115.198	44.705	-1.368	2.023E-02
GAS7	1687.230	669.471	-1.332	5.590E-14
SPINT1	668.964	267.297	-1.321	4.049E-08
P2RX1	128.823	51.655	-1.318	3.371E-02
ABCA8	391.088	160.457	-1.292	7.856E-05
RP4-792G4.2	141.642	58.416	-1.288	2.728E-02
CARD14	212.580	87.699	-1.272	3.514E-03
FRAT1	227.996	94.158	-1.271	2.134E-03
GGACT	226.929	94.701	-1.258	2.290E-03
CTB-181H17.1	337.465	140.720	-1.257	1.496E-04
DAPL1	200.654	84.272	-1.250	3.213E-03
ZNF114	161.222	67.670	-1.247	1.291E-02
NRG2	247.983	105.997	-1.223	1.791E-03
ZMYND10	184.036	78.723	-1.222	6.497E-03
WNT4	661.109	283.870	-1.220	2.664E-08
TMEM238	366.973	157.629	-1.212	1.680E-03
AC112198.1	121.660	52.815	-1.203	3.708E-02
LRRC32	2510.978	1094.835	-1.198	4.218E-29
PRSS56	145.473	63.538	-1.197	2.308E-02
GUCY1A2	142.908	62.716	-1.195	2.852E-02
AC002429.5	269.401	119.832	-1.171	3.553E-03
TMCC2	234.532	105.417	-1.151	1.290E-02
P2RY6	553.376	250.226	-1.148	1.548E-06
ARVCF	309.757	142.173	-1.120	8.429E-04
RP11-347E10.1	188.970	87.457	-1.110	2.321E-02
FMN1	193.095	89.982	-1.099	1.171E-02
SYTL5	741.815	349.442	-1.088	1.700E-07
FCGRT	302.830	142.893	-1.086	1.337E-03
GPAT2	750.965	355.328	-1.082	1.805E-07
RNF43	210.312	98.825	-1.082	2.514E-02
PRRG3	379.392	178.753	-1.081	1.185E-03
ITGA7	359.181	169.447	-1.080	5.209E-04
BZRAP1	301.570	145.904	-1.055	3.051E-02
A2M	18215.064	8803.670	-1.049	4.365E-100

CYSLTR2	421.822	203.766	-1.048	2.023E-04
TIAM1	283.715	137.630	-1.044	2.345E-03
SEPT5	258.308	125.506	-1.044	6.586E-03
MMRN2	236.062	114.400	-1.043	1.585E-02
GXYLT2	2723.661	1330.526	-1.033	1.902E-18
CRYAB	3013.993	1475.219	-1.031	1.852E-27
USH1C	3467.462	1712.602	-1.019	2.543E-27
GPER1	188.831	93.747	-1.007	2.813E-02
SLC38A3	222.368	111.480	-1.001	1.895E-02
EEF1A1P11	483.337	974.941	1.014	1.658E-08
RPS3AP5	294.073	594.065	1.016	1.379E-05
RP11-159J3.1	632.804	1282.166	1.020	9.629E-12
POLR3G	539.883	1109.279	1.039	7.663E-10
RP11-215A21.2	80.661	168.947	1.069	2.798E-02
RPS26P3	130.153	274.416	1.076	3.307E-03
HMGB1P10	167.752	356.618	1.089	3.340E-04
MYEOV	62.344	133.704	1.100	4.585E-02
HMGN2P17	78.463	167.736	1.100	3.777E-02
GAPDHP21	96.415	207.672	1.106	7.818E-03
ACTA1	57.816	127.076	1.135	4.186E-02
SERPINE2	5675.867	12503.478	1.139	2.972E-111
CXCL1	123.755	275.297	1.155	1.933E-03
UBASH3B	190.932	440.274	1.205	9.963E-06
SLC16A9	232.427	541.385	1.219	3.601E-07
RP11-475J5.8	76.531	178.446	1.222	8.069E-03
CH17-12M21.1	450.001	1073.311	1.255	1.295E-12
AC090602.2	47.890	114.481	1.260	3.729E-02
RP11-159H3.1	91.086	220.413	1.275	1.287E-03
FTH1P4	472.948	1161.995	1.298	1.198E-15
RP11-553P9.1	92.218	229.880	1.319	7.579E-04
HSPE1P25	41.863	111.714	1.416	1.812E-02
TNPO1P1	30.506	85.447	1.483	4.916E-02
POTEI	109.836	314.974	1.521	4.755E-06
TUBB8	30.273	88.339	1.542	2.919E-02
DHDH	38.465	117.674	1.616	8.376E-03
TUBB8P7	25.078	79.303	1.662	2.405E-02
CMTM7	24.079	77.865	1.691	2.516E-02
KBTBD11	22.912	82.070	1.847	1.773E-02
IL8	18.883	73.234	1.953	1.632E-02
EEF1A1P25	22.447	91.891	2.030	3.914E-03
RP1-72A23.3	55.819	231.700	2.051	4.835E-06

Supplementary Table 3

Differentially expressed genes in 20:1 sample vs untagged control (CTRL).

gene	Mean counts (10:1)	Mean counts (CTRL)	Log2FoldChange	p_adj
NWD1	101.526	9.970	-3.333	6.168E-05
CALHM3	86.927	12.008	-2.865	5.411E-04
SEZ6	86.367	16.394	-2.404	1.770E-03
HIST1H4A	139.558	28.205	-2.311	7.943E-05
FILIP1	102.014	21.324	-2.268	8.273E-03
TPT1P1	109.838	22.909	-2.259	6.828E-04
AC112198.1	127.578	28.395	-2.163	3.656E-04
P2RX1	135.086	30.594	-2.136	1.040E-02
ALPI	273.145	62.086	-2.134	6.977E-07
ESAM	89.825	21.217	-2.078	5.183E-03
PADI2	122.619	30.680	-2.003	8.636E-04
FOLH1	105.507	26.435	-1.991	4.282E-03
TAF7L	83.854	21.118	-1.983	1.463E-02
CCL8	85.111	21.732	-1.965	9.379E-03
RBP1	139.033	36.363	-1.937	3.902E-04
RP11-991C1.1	145.041	38.266	-1.916	1.604E-03
GAS7	1769.293	469.418	-1.916	4.878E-26
DLK2	105.402	28.255	-1.903	4.831E-03
LGSN	402.153	107.770	-1.896	1.423E-06
TMEM45B	299.650	81.371	-1.880	9.665E-08
ARC	169.662	46.248	-1.869	9.887E-03
ODF3L1	92.619	25.335	-1.869	5.666E-03
RP4-792G4.2	148.533	40.770	-1.863	3.550E-04
LRRC73	89.127	25.236	-1.819	1.005E-02
SCEL	127.300	36.059	-1.813	1.398E-02
TJP3	166.869	48.965	-1.774	1.913E-03
RP11-635N19.3	74.738	21.831	-1.773	2.393E-02
ADAMTS2	162.677	48.224	-1.757	4.682E-04
AFF3	177.240	53.673	-1.719	8.078E-03
CBLN3	98.382	29.939	-1.712	1.144E-02
WNT4	693.284	212.292	-1.707	3.391E-14
PRSS56	152.549	48.104	-1.669	2.507E-03
CYSLTR2	442.352	139.396	-1.666	4.066E-09
RP11-344E13.3	289.172	91.222	-1.665	7.539E-06
LINC01132	140.325	44.542	-1.654	8.229E-03
CYP3A5	121.956	38.838	-1.651	7.267E-03
PTP4A3	124.295	39.592	-1.648	6.483E-03

AL139147.1	79.418	25.504	-1.634	3.547E-02
EPHA4	121.675	39.670	-1.618	9.539E-03
COL5A2	285.085	93.528	-1.609	3.788E-05
PDGFB	108.125	35.581	-1.607	1.329E-02
SSC5D	145.496	47.574	-1.607	2.108E-02
RP11-317N8.5	96.951	31.801	-1.604	3.927E-02
CAMSAP3	185.622	61.501	-1.594	4.091E-04
PMEL	129.919	43.562	-1.573	6.084E-03
CCL7	77.148	26.216	-1.556	4.092E-02
TPPP3	314.004	106.826	-1.554	9.811E-06
DAPL1	210.419	71.661	-1.550	1.211E-03
MMP24	132.678	45.840	-1.530	7.983E-03
GUCY1A2	149.860	52.009	-1.522	1.567E-02
IGFBP5	298.778	104.725	-1.512	2.058E-05
C1orf106	232.176	81.668	-1.508	1.310E-04
TIAM1	297.520	105.248	-1.502	5.031E-05
NCCRP1	413.260	148.332	-1.481	2.760E-05
CRISPLD2	378.196	135.525	-1.481	9.127E-07
MMP28	220.930	80.053	-1.466	5.461E-04
RIMBP3	107.952	39.374	-1.453	3.399E-02
LRRC32	2633.151	966.718	-1.445	1.279E-38
BMPER	188.136	69.334	-1.437	4.895E-03
RP11-21G15.1	127.125	47.124	-1.435	1.659E-02
NT5M	172.107	64.293	-1.420	2.541E-03
FOXN1	143.748	54.845	-1.395	2.429E-02
PVRL4	397.823	151.085	-1.394	3.458E-04
LINC00570	95.728	36.582	-1.387	3.558E-02
GAS6-AS1	95.448	36.532	-1.384	3.846E-02
SP6	405.505	155.388	-1.383	5.913E-06
EDAR	162.956	63.123	-1.371	6.155E-03
TMCC2	245.935	95.348	-1.370	3.204E-03
CRYAB	3160.649	1222.645	-1.369	1.075E-27
FOSB	1314.273	510.508	-1.363	8.873E-14
B3GALT4	226.833	88.183	-1.362	6.395E-04
CCDC60	192.468	74.799	-1.360	4.325E-03
OLFML2B	167.567	65.117	-1.360	1.768E-02
HIST2H4A	105.087	41.264	-1.350	3.358E-02
RP11-282K24.3	119.161	46.947	-1.345	2.440E-02
MYLK	255.716	100.727	-1.343	3.450E-04
ZMYND10	192.992	76.265	-1.335	1.063E-02
CSPG5	223.271	88.705	-1.334	1.360E-03

FBLN7	377.215	152.224	-1.312	3.306E-04
PRRG3	397.859	161.926	-1.299	9.599E-05
RP3-437C15.1	121.851	49.591	-1.296	2.465E-02
ADAMTS8	339.743	138.619	-1.291	8.287E-04
ALPP	168.650	69.074	-1.288	6.264E-03
SV2A	342.153	140.660	-1.285	4.451E-04
REPS2	275.973	113.214	-1.282	2.621E-03
FOXS1	377.672	155.424	-1.282	1.801E-05
OTOG	213.807	88.055	-1.277	3.611E-03
KRT13	297.276	122.804	-1.274	2.458E-04
ZNF329	167.811	69.688	-1.269	8.679E-03
ARVCF	324.831	135.018	-1.268	2.235E-04
WNT11	676.307	282.676	-1.258	1.022E-07
TMEM238	384.831	160.774	-1.257	9.148E-05
ATP2A3	2376.844	995.095	-1.255	1.333E-18
GMDS-AS1	222.992	93.634	-1.254	6.028E-03
CITF22-92A6.1	140.430	59.054	-1.252	1.934E-02
FAM179A	329.825	138.520	-1.248	1.674E-03
ELN	265.285	111.854	-1.246	5.667E-04
P2RY6	580.302	244.699	-1.245	2.558E-07
SPINT1	701.527	297.649	-1.238	7.058E-07
NRG2	260.046	110.718	-1.230	2.606E-03
INSL4	809.544	346.031	-1.225	2.210E-08
KRT42P	225.294	96.538	-1.223	3.239E-02
CA11	140.116	60.599	-1.212	2.689E-02
SLC38A3	233.190	101.283	-1.200	6.578E-03
DMTN	211.605	92.095	-1.198	1.371E-02
RP11-127L20.3	160.862	70.293	-1.192	2.364E-02
Z98049.1	148.183	64.829	-1.191	2.812E-02
TSPAN18	1319.059	578.934	-1.187	3.203E-12
A2M	19101.438	8395.962	-1.186	1.465E-10
TMTC2	151.886	66.987	-1.179	2.466E-02
USH1C	3636.177	1609.389	-1.176	1.332E-35
DENND6B	389.230	172.564	-1.173	1.479E-04
PDZK1IP1	153.004	68.045	-1.168	3.137E-02
RP1-193H18.2	128.871	57.411	-1.167	4.647E-02
ACTG2	231.862	103.753	-1.163	5.954E-03
TLE2	396.671	177.452	-1.161	8.990E-05
WISP2	11827.699	5288.938	-1.161	1.768E-59
NYNRIN	665.936	298.676	-1.156	2.865E-07
GPAT2	787.509	355.143	-1.149	7.517E-09

KIF13B	171.548	77.570	-1.143	1.818E-02
IGF1	422.340	191.548	-1.142	1.096E-04
UGT3A1	238.777	108.271	-1.140	5.283E-03
HNRNPLP2	295.914	134.652	-1.138	1.756E-03
MUC16	12893.501	5874.357	-1.134	3.456E-39
RP11-445H22.4	208.952	95.291	-1.133	6.660E-03
KCNK6	210.977	96.213	-1.131	1.191E-02
KRT14	1617.936	740.252	-1.127	2.328E-14
ECEL1	20456.261	9402.903	-1.121	2.970E-91
TMEM217	266.299	122.820	-1.119	8.446E-03
ANO2	491.628	228.460	-1.106	4.433E-05
RP11-290F20.3	227.077	106.178	-1.099	1.240E-02
GPR153	628.954	294.620	-1.092	9.424E-05
NIPAL4	468.472	220.323	-1.088	2.649E-04
JUN	12172.925	5726.404	-1.088	2.508E-54
LRRC26	240.873	113.645	-1.084	4.655E-03
KANK4	1415.586	668.388	-1.083	1.340E-13
RASD1	373.830	176.690	-1.081	2.913E-04
RP11-496N12.6	136.379	64.857	-1.072	4.687E-02
CHST13	764.039	363.873	-1.071	1.797E-07
PRKCB	4476.527	2137.856	-1.066	3.367E-35
OLFML2A	5083.266	2431.070	-1.063	2.604E-24
ADAM19	229.139	110.005	-1.056	2.093E-02
TM4SF1-AS1	324.481	156.100	-1.055	1.651E-03
ITGA11	4325.896	2086.461	-1.052	2.635E-31
PRSS36	717.902	347.583	-1.046	3.364E-06
GXYLT2	2856.220	1383.154	-1.045	7.394E-16
CTB-181H17.1	353.888	172.099	-1.039	1.227E-03
SYCE1L	158.451	77.232	-1.037	3.596E-02
C1orf116	235.040	114.851	-1.035	1.261E-02
GDPD5	1519.970	744.263	-1.031	1.170E-07
FAM189A2	249.325	121.985	-1.028	2.021E-02
AC002429.5	282.501	138.692	-1.028	1.180E-02
FBLN5	14497.778	7117.839	-1.026	2.908E-44
F10	162.467	79.876	-1.024	4.419E-02
CD74	6046.164	2981.848	-1.019	1.978E-38
TM7SF2	1380.173	681.086	-1.018	1.187E-11
RP11-469M7.1	242.619	120.449	-1.009	1.087E-02
AC022210.2	145.843	292.704	1.004	6.131E-03
OSGIN1	3308.277	6678.249	1.013	2.865E-02
TGFA	92.096	186.746	1.020	3.615E-02

CYCSP55	150.908	306.249	1.021	2.878E-03
SERPINE2	5952.043	12085.128	1.022	1.640E-32
STC2	1953.381	3981.172	1.027	1.211E-03
ABCC2	123.805	254.037	1.034	3.831E-02
HSPD1P6	146.683	300.820	1.038	4.917E-03
GAPDHP21	101.106	208.083	1.041	1.389E-02
RP11-159J3.1	663.597	1371.013	1.047	2.868E-11
EEF1A1P4	77.217	160.366	1.054	3.264E-02
RPL5P17	101.455	212.123	1.062	1.677E-02
LYPD6B	75.856	159.204	1.069	3.647E-02
RP11-613M5.2	84.517	177.247	1.069	2.106E-02
HMGB1P10	175.914	371.007	1.077	4.045E-04
MRPL3P1	153.912	325.225	1.080	1.221E-03
RP1-95L4.4	72.782	156.622	1.104	3.593E-02
FOSL1	478.636	1041.850	1.121	7.664E-07
HMGN2P17	82.282	179.363	1.125	1.581E-02
PTGES3P3	95.902	212.341	1.146	5.593E-03
GCLM	17602.932	39016.937	1.148	1.560E-06
EPGN	400.686	890.538	1.151	7.191E-06
CH17-12M21.1	471.897	1049.225	1.153	9.827E-12
AKR1C1	4007.531	9003.367	1.168	6.352E-06
UBASH3B	200.221	455.137	1.184	1.349E-05
HTRA3	2500.998	5686.124	1.185	2.082E-04
SLC16A9	243.737	554.868	1.187	1.510E-06
CXCL1	129.779	296.045	1.192	2.224E-03
AKR1B10	226.623	532.231	1.231	7.871E-06
GCLC	4812.433	11356.236	1.239	2.000E-04
PCDH1	58.847	140.483	1.252	4.994E-02
RP11-288H12.4	61.641	147.411	1.259	3.542E-02
RP11-159H3.1	95.518	237.050	1.310	1.016E-03
LINC01094	53.574	132.590	1.311	4.481E-02
RNF144B	46.763	118.829	1.342	4.373E-02
RPS4XP3	34.854	89.529	1.361	4.945E-02
CTTNBP2	51.933	138.013	1.413	1.349E-02
AKR1C2	43218.820	115245.605	1.415	8.852E-10
RP11-553P9.1	96.705	260.014	1.428	1.258E-04
LINC00942	60.524	169.676	1.485	5.238E-03
TNIP3	32.934	93.541	1.508	3.452E-02
POTEI	115.180	328.891	1.514	3.575E-06
RP11-20O24.1	30.454	87.190	1.517	4.052E-02
TUBB8P7	26.298	76.697	1.543	4.225E-02

RP1-72A23.3	58.533	172.856	1.559	4.282E-03
AP003068.18	33.527	98.964	1.563	1.678E-02
RPL36AP13	39.604	117.792	1.573	9.301E-03
FTH1P4	495.960	1480.535	1.577	3.689E-22
HSPE1P25	43.900	144.424	1.717	1.141E-03
NDUFS5P3	25.914	89.431	1.789	1.085E-02
MT1F	81.619	291.396	1.837	3.697E-05
EEF1A1P25	23.539	86.004	1.866	1.140E-02
DHDH	40.338	154.415	1.940	2.940E-04
MT1H	25.914	99.939	1.947	9.718E-03
HMOX1	8960.776	42352.943	2.241	1.268E-05
MT1G	9.569	102.640	3.419	4.954E-05
